# Interspecific Tests of Allelism Reveal the Evolutionary Timing and Pattern of Accumulation of Reproductive Isolation Mutations

**DOI:** 10.1371/journal.pgen.1004623

**Published:** 2014-09-11

**Authors:** Natasha A. Sherman, Anna Victorine, Richard J. Wang, Leonie C. Moyle

**Affiliations:** 1 Department of Biology, Indiana University, Bloomington, Indiana, United States of America; 2 Laboratory of Genetics, University of Wisconsin, Madison, Madison, Wisconsin, United States of America; University of California, Berkeley, United States of America

## Abstract

Despite extensive theory, little is known about the empirical accumulation and evolutionary timing of mutations that contribute to speciation. Here we combined QTL (Quantitative Trait Loci) analyses of reproductive isolation, with information on species evolutionary relationships, to reconstruct the order and timing of mutations contributing to reproductive isolation between three plant (*Solanum*) species. To evaluate whether reproductive isolation QTL that appear to coincide in more than one species pair are homologous, we used cross-specific tests of allelism and found evidence for both homologous and lineage-specific (non-homologous) alleles at these co-localized loci. These data, along with isolation QTL unique to single species pairs, indicate that >85% of isolation-causing mutations arose later in the history of divergence between species. Phylogenetically explicit analyses of these data support non-linear models of accumulation of hybrid incompatibility, although the specific best-fit model differs between seed (pairwise interactions) and pollen (multi-locus interactions) sterility traits. Our findings corroborate theory that predicts an acceleration (‘snowballing’) in the accumulation of isolation loci as lineages progressively diverge, and suggest different underlying genetic bases for pollen versus seed sterility. Pollen sterility in particular appears to be due to complex genetic interactions, and we show this is consistent with a snowball model where later arising mutations are more likely to be involved in pairwise or multi-locus interactions that specifically involve ancestral alleles, compared to earlier arising mutations.

## Author Summary

The evolution of reproductive barriers between species, like inviability and sterility in hybrids, continues to fascinate and puzzle evolutionary biologists. However, very few studies have successfully identified the genes responsible for these barriers, or when the underlying mutations appeared during species' evolutionary history of divergence. Differentiating whether specific isolation-causing mutations evolved early versus late in the divergence history of lineages can reveal important insights into the mechanisms of speciation—how new species are formed. Here we infer the evolutionary timing of these loci using data on the chromosomal location of genes that cause reduced hybrid pollen and seed fertility among three species in the wild tomato group, and information on their evolutionary relationships. With genetic crosses that combine these sterility loci from different lineages, we evaluate whether sterility effects are due to the same mutational change(s)—earlier in the history of evolutionary divergence among these species—or to independent mutational changes—later in their evolutionary divergence. We show that most sterility loci separating species are unique to a single species pair, and most isolation-causing mutations arose on recent evolutionary branches. Our data are consistent with mathematical models that predict that these loci should ‘snowball’ between species as they diverge.

## Introduction

New species evolve via the accumulation of genetic changes that confer ecological differences and, in sexually reproducing organisms, reproductive isolating barriers. Of these barriers, hybrid sterility and inviability are thought to be due to deleterious interactions between mutations, at two or more loci, that have arisen and fixed in diverging lineages (‘Dobzhansky-Muller Incompatibilities’ [DMIs]; [1]–[3]. After several decades of intense empirical interest, the genetic basis of these loci is starting to be understood. In many cases, Quantitative Trait Locus (QTL) mapping has identified chromosomal regions that contribute to the expression of reproductive isolation at specific reproductive stages (e.g. male sterility) among lineages e.g., [4]–[12]. In fewer cases, the molecular loci and specific mutations responsible for the expression of hybrid problems have been identified (reviewed in [13], [14]). Despite these emerging data, most studies have yet to reveal the precise origin and historical accumulation of hybrid sterility and inviability loci across a genome through time. These data are critical for understanding both the evolutionary dynamics of lineage divergence and the underlying genetics of speciation.

Distinguishing the isolation-causing mutations that arose early between diverging lineages is essential for identifying mechanisms that predominate during speciation itself, rather than after divergence is substantially complete. Whether incompatibilities that arise early versus later in species divergence have different genetic properties (e.g., effect sizes, kinds of mutational change, and classes of genes) or evolutionary dynamics remains unknown. Understanding the historical origin of isolation loci among species across a whole genome also enables empirical tests of analytical predictions about the temporal accumulation of these mutations, and the nature and complexity of their underlying genetic interactions [15]–[18]. For example, mathematical theory predicts that isolation-causing interactions (DMIs) should accumulate at a pace that is faster than linear with time (i.e., the ‘snowball effect’, [15])– a prediction that requires data on the number of isolation loci in species pairs that span a range of divergence times [3]. Similarly, patterns of incompatibility sharing across species pairs can be used to estimate the relative frequencies of different types of incompatibilities, including derived–derived versus derived–ancestral incompatibilities [19] (and see below). These data in turn enable the dynamics and genetics of reproductive isolation to be compared with other well-understood evolutionary processes, such as adaptation [20].

Empirical studies of the evolutionary accumulation of isolation loci are currently limited to two approaches; individually, neither approach is able to assign the origin of all known reproductive isolation loci in a genome to specific evolutionary branches of a phylogenetic tree. First, some studies have inferred the history of nucleotide changes in individual genes known to contribute to reproductive isolation (reviewed in [13]). These analyses have revealed patterns consistent with repeated nucleotide substitutions at the target locus, in some cases in more than one lineage, e.g., [21]–[26]. Such studies can identify evolutionary branches along which many or most of these mutations have occurred. However, because the specific molecular loci underlying reproductive isolation phenotypes are unknown in most cases, these analyses remain uncommon. Moreover, they cannot assess genome-wide patterns of isolation accumulation among species because they are almost always limited to the analysis of single loci.

Alternatively, other studies have examined the genome-wide pattern of accumulation of all detectable loci contributing to reproductive isolation (i.e. DMIs), using QTL data from two or more species crosses from the same closely related group, e.g., [27]–[29]. Such comparative QTL data can evaluate general patterns of evolutionary accumulation of DMIs with genetic distance (e.g., the ‘snowball effect’; [30], [31]) or the classes of genetic interactions that underpin reproductive isolation, e.g. [32]. However, these analyses do not assign individual isolation mutations to particular evolutionary branches, and therefore cannot address the order in which specific causal mutations arose during the history of divergence between species.

One alternative to these approaches is to combine information from comparative mapping data, and phylogenetic relationships among species involved in these mapping experiments, to infer the order and timing of evolutionary changes responsible for reproductive isolation [20]. Comparative mapping in different species pairs can reveal two classes of isolation QTL: those that are unique to individual species crosses, and those localized to the same chromosomal region in more than one species pair (co-localized/shared QTL). Loci unique to a single species pair must have evolved along evolutionary branches that are unique (unshared) among the species in the comparison; in contrast, a QTL that is shared among multiple species pairs must involve a genetic change along an evolutionary branch that is shared by all species involved in the comparison [20], [30] (Figure S1). Although this reasoning is straightforward, inferences from ‘shared’ reproductive isolation loci rely on the assumption that these co-localized QTL are evolutionarily homologous, that is—they are caused by the same underlying mutational change. However, because QTL regions frequently span multiple genes, QTL co-localization could also occur because different isolation-causing mutations happen to occur in close proximity [27]. To draw strong inferences about evolutionary events from comparative mapping data, therefore, requires experiments that directly assess whether co-localized sterility QTL are indeed homologous or not.

**Figure 1:**
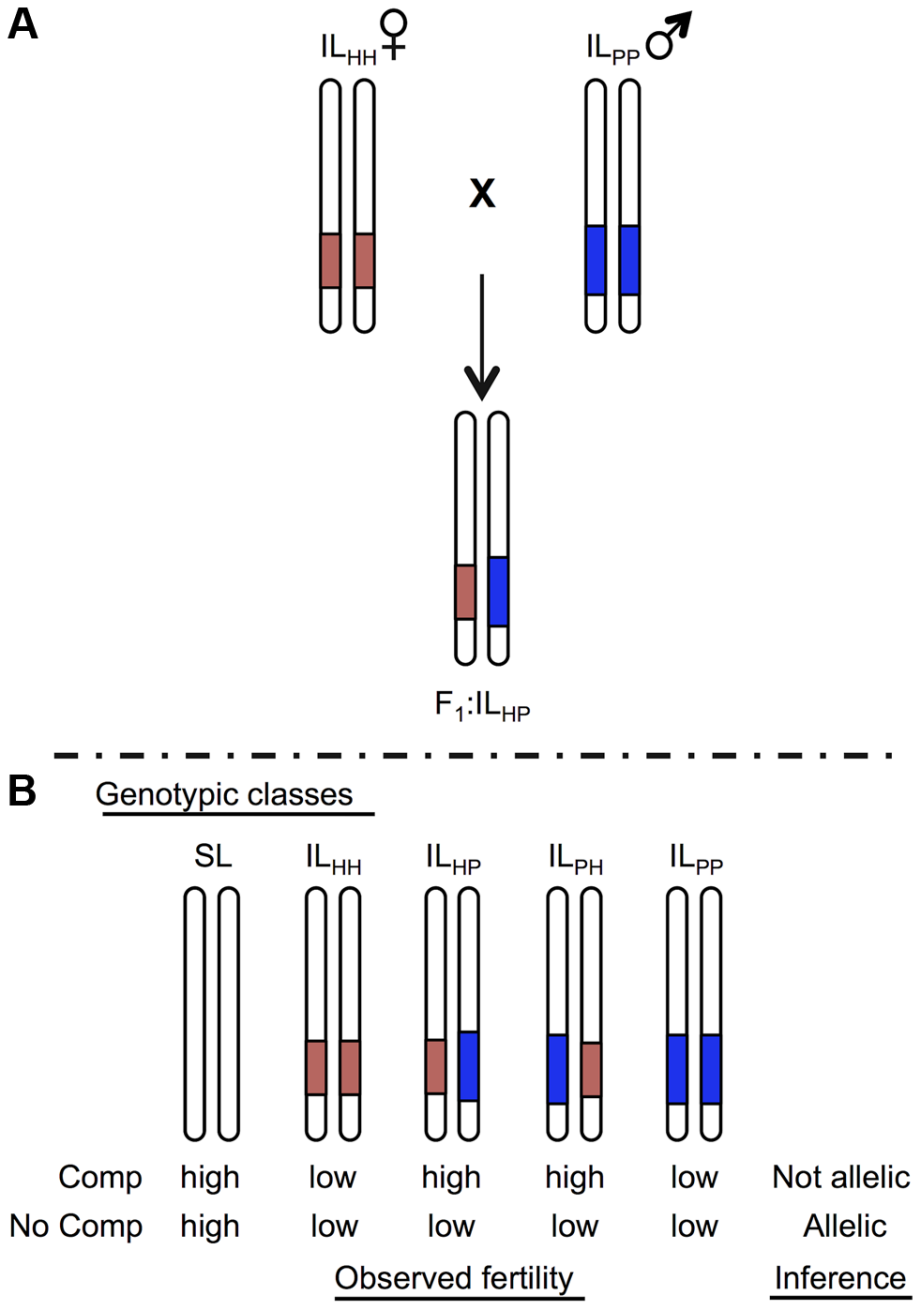
Schematic of a cross species test of allelism to determine homology at co-localized hybrid sterility loci. For illustration, only the chromosome containing the target locus is shown for each genotype. Introgression lines (ILs) show locations of the heterospecific chromosomal region containing an isolation QTL in red (light) shaded and blue (dark) shaded from species H and P, respectively, on a species SL (white) genetic background. A) Homozygous ILs are crossed together to generate heterointrogression lines IL_HP_ or IL_PH_. B) Fertility of hybrid heterospecific ILs are compared with homozygous parental ILs and the pure (fertile) parent to determine evidence for homology. Complementation indicates that alleles are not homologous; lack of complementation (low fertility in the heterointrogression line(s)) is consistent with a shared underlying mutation.

Whether mutations are identical (i.e. occur in the same locus) can be experimentally evaluated by testing for genetic complementation when combined via crossing. Analogous ‘tests of allelism’ can be performed to assess whether co-localized QTL are homologous, e.g., [33], as long as these loci act recessively. In the case of co-localized (and recessively acting) sterility QTL from different species pairs, if a hybrid that combines both QTL shows restored (rescued) fertility, the underlying mutation(s) are inferred to be different because sterility has been complemented (Figure 1). Conversely, when fertility remains low in hybrids, either the underlying genetic lesion occurs in the same locus or additional interactions between these loci contribute to retaining low hybrid fertility (see Results and Discussion). Cross-species tests of allelism can therefore clarify whether co-localized QTL are homologous, without requiring specific knowledge of the underlying molecular lesions.

We have previously mapped QTL for hybrid pollen and/or seed sterility in several interspecific crosses among *Solanum* species [7], [27], [30]. Because of karyotypic co-linearity and common markers between these species, we can delineate chromosomal regions in which a QTL for the same sterility trait appears in more than one species pair [27]. In this study, we evaluate evidence for homology at three such co-localized hybrid sterility QTL using tests of allelism, and find different histories for each locus. With these data, and data on QTL that are unique to each species pair, we infer the timing and pattern of accumulation of all known sterility-causing mutations among these three species. We then evaluate the consistency of these findings with current theory on the evolutionary accumulation of reproductive isolation genes, including incompatibilities based on interactions between loci that have sequentially fixed along a lineage and/or that involve multi-locus interactions. In addition to examining the properties of earlier versus later fixing mutations, our data supports models of non-linear (‘snowballing’) accumulation of the loci underlying both seed and pollen sterility, although the details of this non-linear accumulation differ between the two postzygotic isolation traits. Interpreting these findings in light of theoretical predictions, our results suggest that these traits are underpinned by different classes of genetic interaction, and possibly fixed by different evolutionary dynamics.

## Results

### Tests of allelism between co-localized hybrid sterility QTL

**Figure 2:**
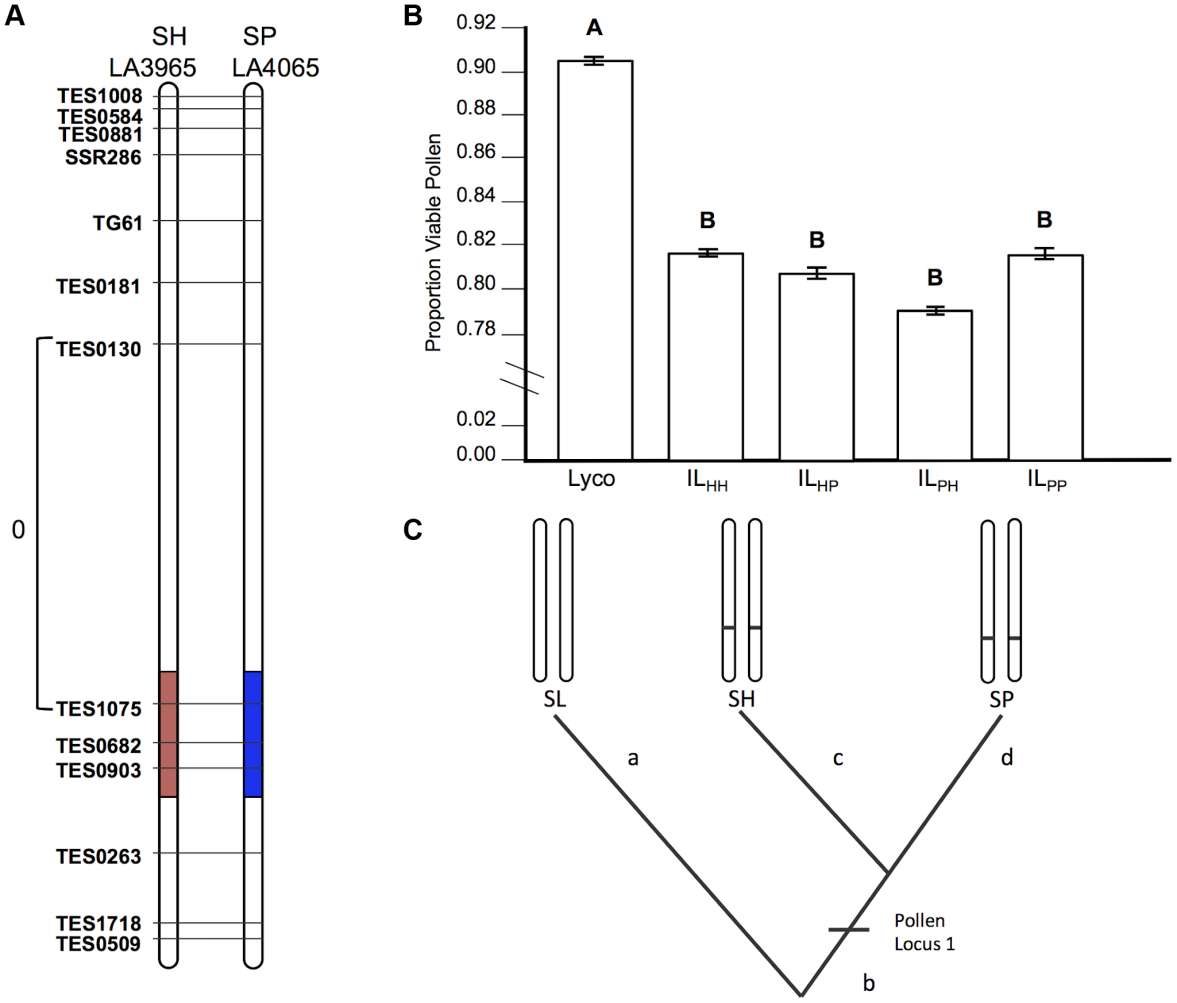
Test of allelism at pollen sterility locus *pf7.2*. A) Chromosomal location (shaded) of SP and SH introgressions represented in IL_PP_ and IL_HH_ lines, respectively, used in cross-species tests. Marker IDs (solgenomics.org) are shown to the right (SP) or left (SH) of chromosome 7. To resolve the chromosomal position of each introgression boundary more finely than in the original mapping studies, we performed additional fine-scale genotyping of IL_PP_ and IL_HH_ lines (Text S1); marker genotypes are from these analyses. B) Pollen fertility (percent fertile pollen) in 5 genotypes (SL = *S. lycopersicum*; IL_HH_ = introgression line with homozygous SH alleles; IL_PP_ = introgression line with homozygous SP alleles; IL_HP_ = heterointrogression line (from IL_HH_ maternal parent); IL_PH_ = heterointrogression line (from IL_PP_ maternal parent)). C) Inferred placement of mutational change underlying *pf7.2*.

**Figure 3:**
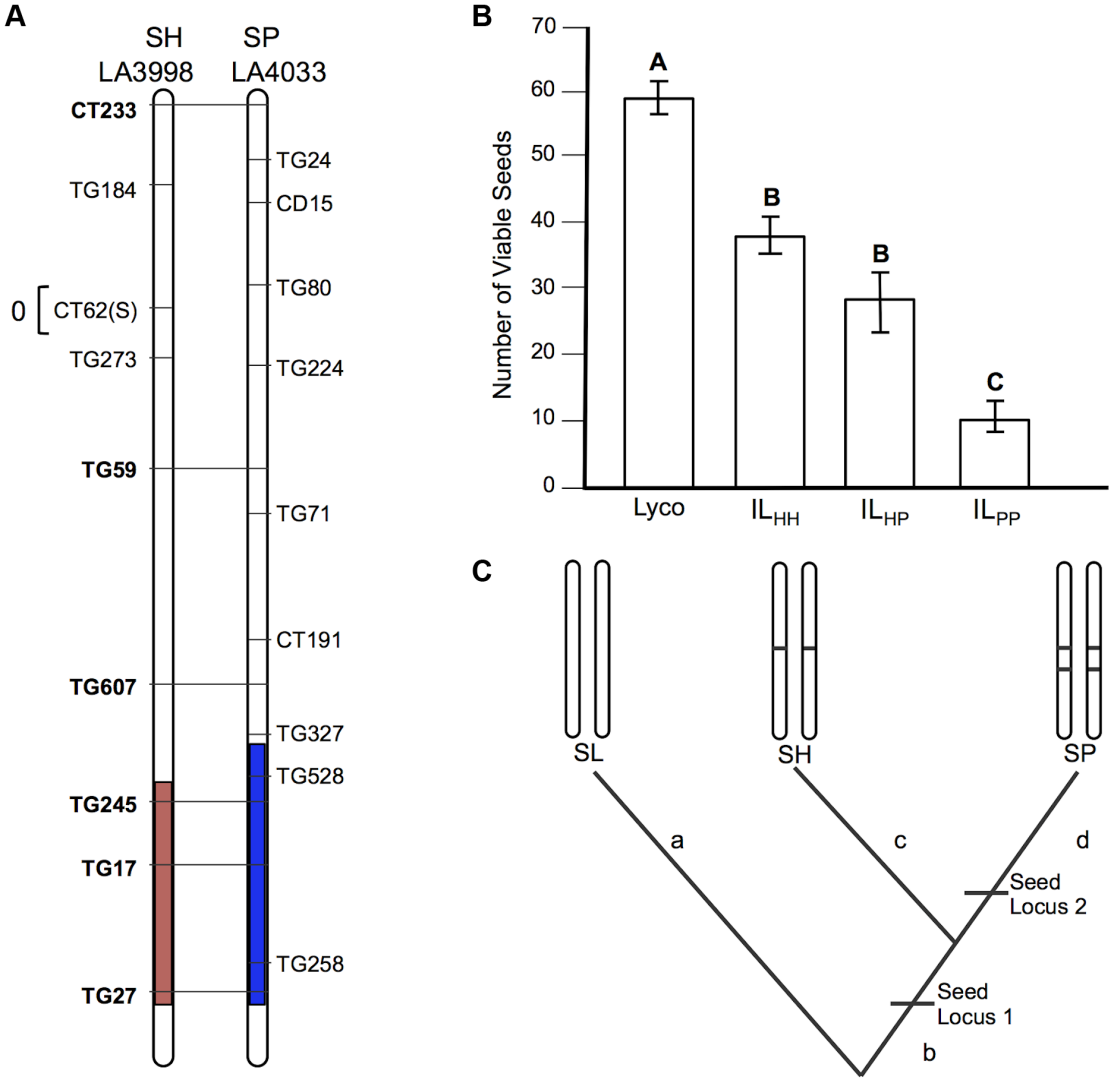
Test of allelism at seed sterility locus *sss1.2*. A) Chromosomal location (shaded) of SP and SH introgressions represented in IL_PP_ and IL_HH_ lines, respectively, used in cross-species tests. Marker IDs (solgenomics.org) are shown to the right (SP) or left (SH) of chromosome 7. B) Seed fertility (number of viable seed per fruit) in 4 genotypes (SL = *S. lycopersicum*; IL_HH_ = introgression line with homozygous SH alleles; IL_PP_ = introgression line with homozygous SP alleles; IL_HP_ = heterointrogression line (from IL_HH_ maternal parent)). C) Inferred placement of two mutational changes (*sss1.2.1*, *sss1.2.2*) underlying *sss1.2*. Note that introgression regions corresponding to *sss1.2* in the SP versus SH lines are incompletely overlapping (Fig. 2A), with an additional distal heterospecific region represented in line IL_PP_ but not in IL_HH_; therefore *sss1.2.2* could be located within the introgressed region unique to IL_PP_. Regardless, because no IL_HH_ line exhibited reduced seed fecundity at this genomic location in the original SH×SL mapping experiment [7], our results are consistent with the inference that *sss1.2.2* is SP-specific.

**Figure 4:**
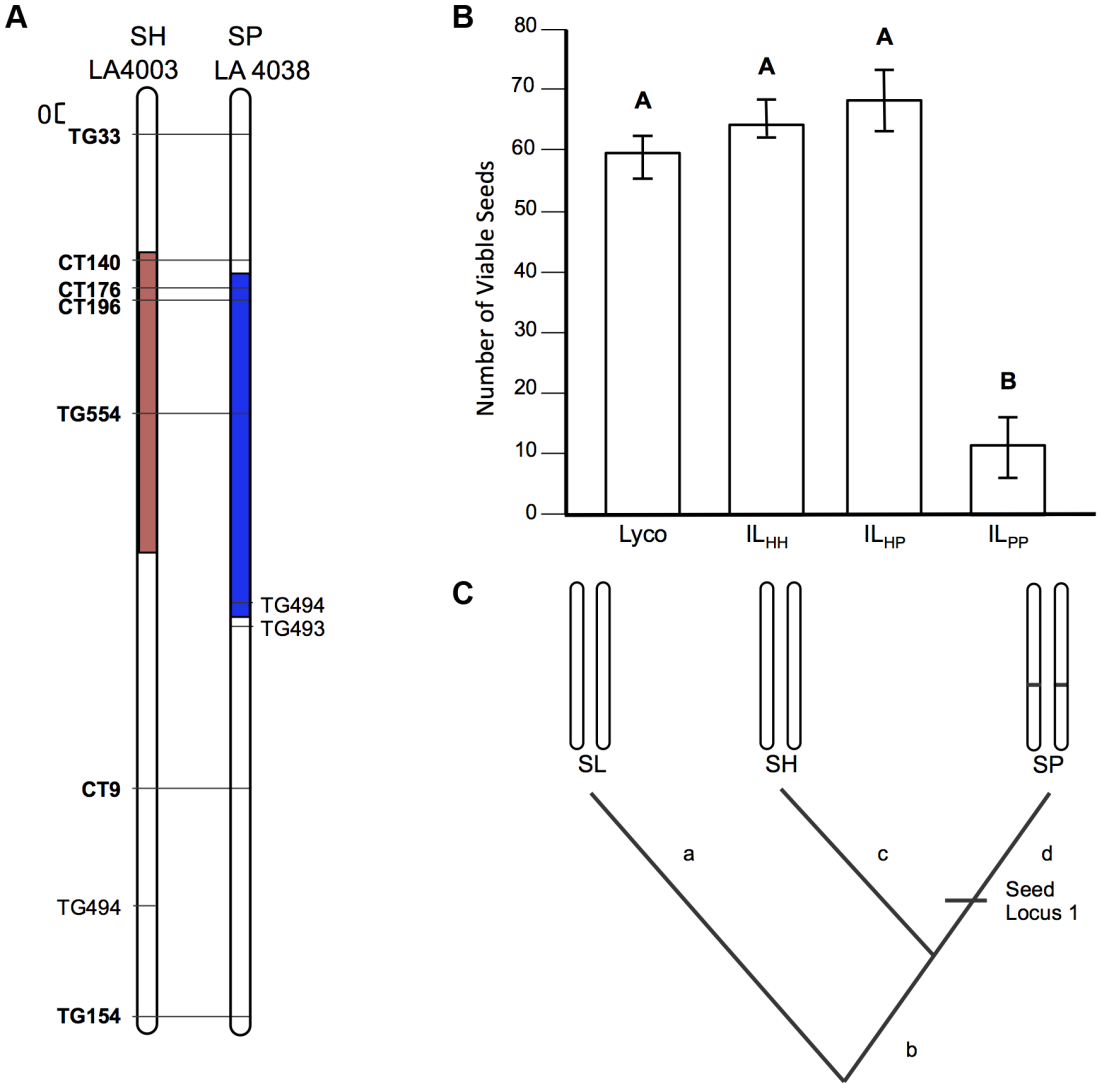
Test of allelism at seed sterility locus *sss2.1*. A) Chromosomal location (shaded) of SP and SH introgressions represented in IL_PP_ and IL_HH_ lines, respectively, used in cross-species tests. Marker IDs (solgenomics.org) are shown to the right (SP) or left (SH) of chromosome 7. B) Seed fertility (number of viable seed per fruit) in 4 genotypes (SL = *S. lycopersicum*; IL_HH_ = introgression line with homozygous SH alleles; IL_PP_ = introgression line with homozygous SP alleles; IL_HP_ = heterointrogression line (from IL_HH_ maternal parent)). C) Inferred placement of mutational change underlying *sss2.1*.

Our analysis examined loci contributing to reproductive isolation among three species in the plant genus *Solanum* (*Solanum lycopersicum* (SL), *S. pennellii* (SP), and *S. habrochaites* (SH)), whose phylogenetic relationships are well understood (Figures 2–4 and Text S1). Previous QTL mapping experiments in crosses between SL and SH [7] and between SL and SP [27] identified 8 and 7 QTL, respectively, associated with hybrid pollen sterility, and at least 4 and 4 QTL, respectively, associated with hybrid seed sterility (Table S1). In both mapping experiments, QTL were detected using introgression lines (ILs), where short chromosomal regions from a donor species (either SH or SP) were introgressed into an otherwise isogenic recipient species (SL) background. Individual ILs in which fertility is significantly reduced are inferred to contain an allele (QTL) from the donor species that causes sterility when interacting with the recipient species' genetic background. Therefore, each QTL corresponds to one side of a DMI (the donor allele from either SP or SH); these experiments are not able to identify the location of the SL locus (or loci) with which this allele is interacting.

Of the sterility QTL detected in these two experiments, three QTL appeared to be chromosomally co-localized (Table 1): one associated with reduced pollen fertility (*pf7.2*) and two with reduced seed fertility (*sss1.2*, *sss2.1*). All three loci act fully or partially recessively (Table 1). Although true complementation tests conventionally require that loci are fully recessive [34], complementation tests can be successfully implemented for incompletely recessive loci when the heterozygous phenotype can be distinguished from the homozygous recessive phenotypes [35] as is the case for our three loci (Text S1). For partially recessive sterility loci, the expectations are equivalent to a conventional test of allelism (Figure 1): in the absence of complementation, fertility of the trans heterozygote will be indistinguishable from the (low fertility) homozygous recessive parental lines; in contrast, complementation is indicated by rescued fertility in the trans heterozygote up to at least the fertility of the more fertile heterozygous genotype. For each of these QTL, we crossed ILs that contained the relevant chromosomal region from each of SH and SP (IL_HH_ and IL_PP_ respectively), in an otherwise SL genetic background (Figure 1). We compared the average pollen and seed fertility of the resulting F_1_ SH-SP QTL heterozygotes (i.e., IL_HP_ and IL_PH_), to the pollen and seed fertility of SH or SP QTL homozygotes (IL_HH_ and IL_PP_) and of the isogenic SL parent. For completeness, we assayed both pollen and seed fertility in every line (results for the non-focal fertility measure are discussed in Text S2). At each of the three target loci, we detected a strong genotype effect on fertility (p<0.0001, ANOVA; Table S2 and Text S2). In addition, each locus showed different patterns of fertility in tests of allelism, including no evidence of complementation, complete complementation (fertility restoration), and more complex patterns of fertility response (Table 2 and Table S2). Based on these observations, we can make inferences about the historical accumulation of reproductive isolation mutations at each of these QTL.

**Table 1:** Co-localized QTL evaluated with interspecific tests of allelism.

		Species pair SH×SL	Species pair SP×SL	
Fertility phenotype	QTL name	Est. effect size	Est. effect size	Dominance (D)
seed	*sss1.2*	−89.1	−82.9	−0.39
seed	*sss2.1* *	−74.2	−62.5	−0.71
pollen	*pf7.2* **	−39.3	−37.7	−0.28

Estimated effect size is expressed in terms of the percentage phenotypic change compared to the fertile parent SL (Δ%). Average allelic action is expressed as the degree of dominance D [65] where D = 0 indicates additivity, D = −1 indicates fully recessive effects, and −1<D<0 indicates incomplete (partial) recessivity for sterility.

*In the source SH×SL mapping study [7], seed fertility effects were not independent of co-localized pollen sterility effects at this locus.

**This locus is labeled *pf7.1* in the SP×SL mapping study [27], as it was the only pollen fertility QTL detected on chromosome 7 in that study.

**Table 2:** Genotype fertilities (least squares means) from tests of allelism at the three co-localized QTL.

QTL region	*sss1.2*		*sss2.1*		*pf7.2*	
Fertility trait	seed	HSD	seed	HSD	pollen	HSD
**SL**	60.83	A	60.83	A	0.9	A
**NIL_PP_**	10.41	C	11.08	B	0.81	B
**NIL_HH_**	38.13	B	64.89	A	0.76	B
**NIL_HP_**	26.66	B	67.93	A	0.77	B
**NIL_PH_**	N/A	N/A	N/A	N/A	0.78	B

Contrasts between all means within a test (i.e. for each locus) were corrected for multiple testing (Tukey tests of Honest Significant Difference (HSD)); different letters indicate significantly different means within each locus.

### Alternative evolutionary histories of mutations underlying isolation loci

#### *pf7.2* is a shared (homologous) allele, indicating at least one early sterility-associated substitution

If the same mutation (allele) in SP and SH causes hybrid sterility in a heterospecific SL background, combining these loci will fail to recover fertility because these alleles will not complement each other (Figure 1). Our results for pollen fertility at *pf7.2* are consistent with this expectation (Table 2). Pollen fertility of the transheterospecific QTL line (IL_HP_) was indistinguishable from introgression lines homozygous for SH or SP alleles (IL_HH_ and IL_PP_) (Figure 2B and Table 2). All introgression lines were significantly less fertile than the parental SL genotype (Figure 2B, Table 2 and Table S2). On average, when homozygous on the SL genetic background, the shared *pf7.2* allele causes a 12.4% reduction in the proportion of fertile pollen (Table 2). Our test of allelism therefore indicates that *pf7.2* is allelic in SH and SP, consistent with the causal mutation(s) arising before SH and SP diverged from their most recent common ancestor (Figure 2C, and see below).

#### *sss1.2* is a complex QTL, with both shared (homologous) and lineage-specific mutations, indicating both early and later sterility-associated substitutions

More complex patterns of fertility at *sss1.2* are consistent with a minimum of two evolutionary changes contributing to hybrid seed sterility at this QTL, one of which is an homologous change (shared by both SP and SH) and one of which is unique to a single evolutionary lineage (SP). This can be inferred via several lines of evidence.

First, the SP allele failed to complement sterility associated with the SH allele (compare mean sterility of IL_HP_ and IL_HH_ in Table 2 and Figure 3B); we infer that SH and SP share at least one mutation that causes reduced fertility in IL_HH_ and contributes to a fraction of reduced fertility observed in IL_PP_. When homozygous on the SL genetic background, we estimate this allele (henceforth *sss1.2.1*) causes an average fertility reduction of ∼47% (∼28 seeds/fruit in this experiment) with respect to SL (Table S2).

Second, although the SP allele did not complement sterility associated with the SH allele, the SH allele did partially complement the more severe sterility observed in the IL_PP_ genotype; that is, the fertility of the transheterospecific (IL_HP_) line was indistinguishable from IL_HH_, but was significantly greater than the highly sterile IL_PP_ (Table 2 and Figure 3B). Therefore, we infer that the more severe sterility observed in IL_PP_ is caused by at least one additional SP-specific mutation (henceforth *sss1.2.2*) that arose after SP split from its most recent common ancestor (MRCA) with SH (Figure 2C); at this second position, SH retains a non-sterility allele that is able to complement the SP-specific allele. When homozygous on the SL genetic background, we estimate that the SP allele at *sss1.2.2* causes an additional ∼68% reduction in fertility, in comparison to the IL_HH_ line (assuming sequential additive effects of the two mutations in SP) (Table S3). In total, the two sterility alleles in SP reduce fertility by ∼83% (∼50 seeds/fruit) in comparison to the fertile SL parental species.

#### *sss2.1* is a lineage-specific locus, indicating at least one later sterility-associated substitution

Unlike our two other loci, patterns of fertility at *sss2.1* were consistent with a sterility mutation unique to one lineage (SP). IL_PP_ had severely reduced fertility—approximately 50 seeds/fruit fewer than any other line—while IL_HH_ and IL_HP_ had fertility indistinguishable from the fertile parent species SL (Table 2 and Figure 4B). Restored fertility in IL_HP_ indicates that sterility is due to at least one recessive mutation unique to SP, that is complemented by the SH allele at this locus (Figure 4C). When homozygous on the SL genetic background, we estimate that the SP allele causes an ∼81% reduction in fertility (Table S3).

*sss2.1* was included in this experiment because both original mapping experiments detected a seed sterility QTL at this location. However, because seed sterility is measured after self-fertilization, it can be influenced by male fertility. Previously *sss2.1* was mapped independently of pollen sterility in one population (SP×SL), but this locus co-occurred with and was statistically non-independent of pollen sterility in the other species cross (SL×SH; Table S1). We previously inferred that *sss2.1* in this second population was likely the secondary consequence of high pollen sterility at this locus, which is consistent with our current observation that the IL_HH_ genotype had normal seed fertility. Our data also confirm that the IL_HH_ genotype has significantly reduced pollen fertility (corresponding to *pf2.1* in [7]), whereas the IL_PP_ does not (Table S2). Greater biological replication in the current experiment might have helped de-confound these pollen and seed effects in IL_HH_ (Table S4). Regardless, our results indicate that the allele responsible for reduced seed fertility at *sss2.1* in IL_PP_ is unique to the SP lineage.

### Phylogenetic placement of mutations underlying reproductive isolation

For each of the three apparently co-localized QTL examined here, we can infer when the underlying mutation(s) evolved. Loci that are unique to a single species pair—*sss1.2.2* and *sss2.1*—are inferred to have evolved on branches that are not shared between the two species pairs; therefore, the mutations underlying these two loci are assigned the SP-specific branch—the only branch exclusive to the SP×SL cross in these two pairs (Figures 2C, 3C and 4C).

In comparison, we assign our two inferred homologous alleles—*pf7.2* and *sss1.2.1*—to the branch shared by both SP and SH after their split from their MRCA with SL (Figures 2C and 3C). This placement assumes that the observed sterility effects are the result of mutations that arose (i.e. were derived) along the lineage that gave rise to SP/SH. The alternative is that sterility between SP/SH and SL at these shared loci is due to ‘derived-ancestral’ interactions [15], where the shared SH/SP allele represents the ancestral state at this locus. Such derived-ancestral interactions arise when multiple mutations occur sequentially along one evolutionary branch, so that later derived mutations arise upon a genetic background of loci that have already experienced new (derived) substitutions. For each of our shared QTL to be due to derived-ancestral interactions, both the causal mutation and the mutation at the other locus/loci with which it interacts must occur on the SL-specific branch. However, at least two lines of evidence indicate that this alternative scenario does not describe the evolutionary history of *pf7.2* and *sss1.2.1* (Text S2). In particular, in QTL mapping experiments with two additional species that are more closely-related to SL (Figure S3), we do not detect pollen or seed sterility loci at these chromosomal locations; these other species should also manifest these QTL when crossed to SL, if the causal mutations were derived along the terminal branch leading to SL. The alternative is that SL shares the same derived alleles at both *pf7.2* and *sss1.2.1* with these 2 other species. However, this requires that both substitutions underlying each putative derived-ancestral interaction must have arisen and fixed along an extremely short evolutionary branch shared by these three species, but not by SH or SP (Figure S3), a hypothesis that is less parsimonious than assuming a single derived mutation for each locus along the much longer shared SH/SP branch (Text S2).

**Figure 5:**
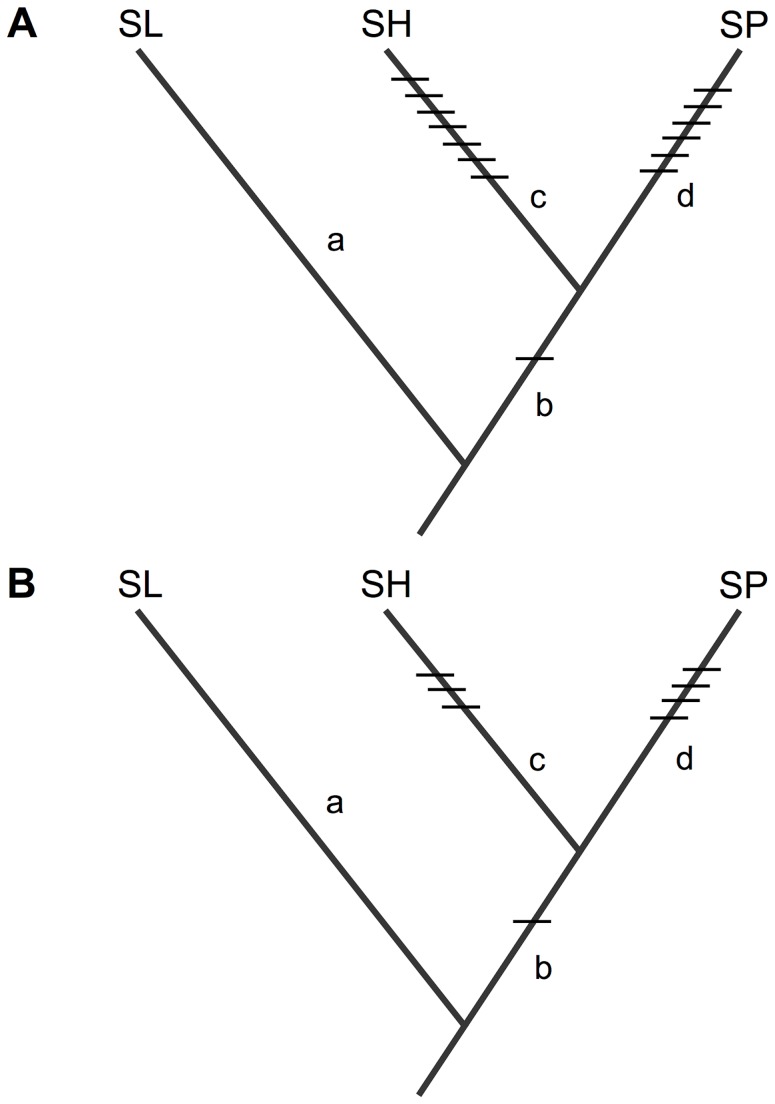
Inferred placement of all detected isolation loci acting on (A) pollen fertility, and (B) seed fertility, between SL and each of SH and SP. Details and effect size data for each locus are in Table S3. Loci that are unique to one species pair must be due to mutations that occurred on a branch that is unique to that species pair. Placement of the shared isolation mutations on branch *b* is based on the inference that they are derived in the common ancestor of SH/SP (see main text, Figures 2 and 3, Text S2, and Figure S3).

In addition to the three co-localized QTL examined in our tests of allelism, earlier mapping studies [7], [27] revealed 13 pollen and 5 seed sterility QTL that were unique to each interspecific cross (Table S1). Using the same logic as for lineage-specific mutations above, mutations underlying these additional pollen and seed sterility QTL can be assigned to either SP or SH terminal branches, depending upon the cross in which the loci were identified. With these data and the results of our tests of allelism, we are therefore able to assign every known sterility locus among these two species pairs to a mutation event on a specific evolutionary branch, conservatively assuming that each QTL is underpinned by one mutation (Figure 5). Finally, note that making general inferences from the resulting patterns of shared versus non-shared isolation loci relies on the assumption that we have not systematically failed to detect co-localized QTL due to low power in our previous mapping experiments. This assumption appears to be reasonable; we find that even with a substantially more permissive statistical threshold for identifying QTL in each original mapping experiment (see Text S1), we do not uncover disproportionately more co-localized QTL than are detected with the more stringent standard cutoffs originally used to identify the QTL analyzed here (Table S8).

### Size, distribution, and pattern of accumulation of sterility-associated mutations over evolutionary time

Our data on the phylogenetic distribution of shared and unique sterility-causing loci (Figure 5) can be used to address several questions about the genome-wide accumulation of mutations that underlie the expression of hybrid sterility. First, we can assess whether ‘earlier’ versus ‘later’ arising mutations differ in their average phenotypic effect size on DMIs. Second, these data can be used to evaluate the temporal distribution of mutations contributing to pollen and seed sterility on our tree, given estimates of relevant branch lengths for this tree. Finally, based on patterns of incompatibility sharing, we can evaluate evidence for alternative models of incompatibility evolution using statistical comparisons in a phylogenetic context [19].

#### Relative effect sizes of DMIs involving earlier versus later substitutions

To compare the phenotypic effect size of the DMI associated with our single ‘early’-arising mutations (one each for pollen sterility and seed sterility) to the effect sizes associated with our ‘late’ mutations, we used a bootstrap approach to re-sample the set of trait (sterility) values associated with our ‘late’ arising mutations (*N* = 500 replicates). For seed sterility loci, the observed ‘early’ effect size was smaller than that of the lower 95% confidence interval (CI) of the simulated distribution of ‘late’ mutation effect sizes (Table S5). In comparison, the effect size of the ‘early’ pollen sterility locus did not consistently fall outside the 95% CI of the simulated distribution of ‘late’ pollen effect sizes (Table S5, Text S2). These findings imply that, for seed sterility, earlier-evolving mutations might result in DMIs with smaller effect sizes than later evolving mutations. However, we have no information about the underlying composition of most of our QTL, especially whether they are underpinned by more than one mutational change; if some of our ‘late’ QTL are polygenic (as we found for 1/3 co-localized QTL, above), they might appear to have larger average effect sizes simply because of the composite effects of >1 mutation. Accordingly, our comparison is fairly permissive with respect to detecting larger effects for ‘late’ QTL. Nonetheless, there is no evidence for the converse inference that earlier substitutions have on average larger sterility effect sizes than later substitutions.

#### Evidence for unequal distribution of sterility-associated mutations over time

Our results also suggest that mutations contributing to DMIs are not equally distributed on early (internal) branches versus late (terminal) branches of our phylogenetic tree; only 1 of 14 pollen, and 1 of 8 seed, sterility QTL are inferred to involve mutations that arose on early branches (Figure 5). We assessed whether the number of observed ‘late’ versus ‘early’ arising mutations was the same or greater than would be expected given the empirical lengths of the branches on which these changes occurred (branches *c* and *d* versus branch *b*; Figure 5), under the null expectation that isolation-causing mutations are distributed in linear proportion to branch lengths. A RAxML [36] ultrametric tree, based on 18 unlinked loci [37] (Figure S3), was used to estimate the relevant branch lengths and their standard deviations, and generate a predicted ratio of (0.251∶0.749) for early∶late mutations under this null expectation (Text S1).

We evaluated whether the observed distribution of ‘early’ versus ‘late’ mutations deviated from this expectation using two approaches. First, χ^2^ goodness-of-fit tests revealed no significant deviation for either seed or pollen sterility loci, although this test approached significance for pollen sterility (Table S6). Second, for seed and pollen datasets separately, we evaluated whether our observed number of ‘late’ mutations was outside the upper 95% CI of a simulated binomial distribution, generated by re-drawing (N = 1000 iterations) the same number of total isolation mutations (trials) as observed in our data (*n* = 8 or 14, for seed and pollen respectively) with the expected probability of a mutation falling on ‘late’ branch determined by our empirical branch lengths (i.e., p_late_ = 0.749). The observed number of late seed sterility loci did not significantly deviate from the binomial simulation results (Table S7), however the observed number of late pollen loci was marginally more extreme (greater) than the simulated distribution; only 8.6% of simulations placed 13 or more (of 14) loci on late branches under this model (Table S7). Although these results are suggestive, because we have relatively few QTL and only a single locus assigned to the ‘early’ branch for both pollen and seed sterility, there is low power to detect significant effects within our dataset (Tables S6 and S7), especially if variance in branch length estimates (specifically overestimation of the relative length of the internal branch) is taken into account (Text S2). Conversely, if we relax our assumption that all unique (late) QTL are underpinned by a single mutation, both methods reject a ‘linear’ distribution of isolation-causing mutations for pollen sterility (Text S2, Tables S6 and S7). An evaluation of whether the more conservative or relaxed assumption is more reasonable will require further fine mapping.

#### Evidence for non-linear accumulation of sterility loci, and complex incompatibilities in pollen sterility

We also evaluated the fit of our data to models that explicitly describe the distribution of isolating incompatibilities along a phylogeny [15], which is the first application of this statistical phylogenetic approach to empirical isolation data. These models are constructed from mathematical descriptions of the incompatibility accumulation process [3], [15], and can be distinguished by the different proportions of shared-to-unshared isolation loci they predict. We compared between four different models of incompatibility accumulation, described in [19]: a linear model (‘linear’), a Dobzhansky-Muller (‘DM’) model restricted to interactions between pairs of loci, a model that allows three loci to participate in DMIs (‘2+3’), and a pairwise DM model that allows derived-derived and derived-ancestral incompatibilities to arise with different probabilities (‘p_a_≠p_d_’). We modified the maximum likelihood method described in [19], specifically by reducing the length and rank of the incompatibility vector and covariance matrix, respectively, to incorporate only the branches from which we have data. To incorporate variability in estimation of the phylogeny, we estimated branch lengths using trees drawn from the posterior distribution of a MrBayes analysis of 18 unlinked loci [37] (Text S1).

Based on a comparison of Akaike Information Criterion (AIC) values calculated for each model, we found that the pollen data supports a DM model of incompatibility accumulation that includes incompatibilities between three loci (2+3 model), and the seed data supports the basic DM model that incorporates only pairwise interactions between isolating loci (DM model) (Pollen – AIC_linear_: 24.1 (*0.2*), AIC_DM_: 20.0 (*0.7*), AIC_p2+p3_: 19.9 (*0.5*), AIC_pa≠pd_: 20.0 (*0.8*). Seed – AIC_linear_: 20.8 (*0.2*), AIC_DM_: 16.6 (*0.4*), AIC_p2+p3_: 17.6 (*0.2*), AIC_pa≠pd_: 17.7 (*0.3*). (Standard deviations for the AIC of each model arising from the variance in phylogeny are in parenthesis). Both sets of data clearly reject the linear model, which had an AIC of at least 3 greater than all other models tested for both pollen and seed sterility.

To examine the statistical power to distinguish between alternative non-linear models, we simulated the number of incompatibilities that would accumulate in the DM, 2+3, and p_a_≠p_d_ models on a random sample of the ultrametrized distribution of MrBayes trees. The parameters of the simulations were set so that the expected number of incompatibilities would match either the pollen or seed datasets respectively. Predictive value was attributed when the model with the minimum AIC matched the ground truth simulation, i.e. when the incompatibility model underlying the simulation was correctly selected as the best fit. These simulations showed that the result from our pollen data—support for the 2+3 model—is substantially more likely to be rejected incorrectly than accepted incorrectly (Precision: 0.53, Recall: 0.10). That is, although we have relatively few loci to fit such models here, and variance in the phylogeny influences the ability to distinguish more complex models from the basic DM non-linear model, simulations suggest that our inference that pollen sterility data support a more complex model is robust. In contrast, support for the basic DM model from the seed data occurs in most of the simulations regardless of the ground truth model (Precision: 0.34, Recall: 0.91) (Text S1).

## Discussion

Understanding the timing and pattern of accumulation of reproductive isolation loci is critical for understanding which sterility-affecting mutations were fixed during earlier stages of divergence between species, and for evaluating general mutational and evolutionary mechanisms responsible for this process. To date, resolving these questions has been challenging because it requires knowledge of loci that contribute to reproductive barriers between species, and a method of identifying the timing of the relevant genetic changes at these loci. Using a combination of comparative mapping, known species relationships, and empirical tests of allelism, here we have inferred the evolutionary timing of mutations underlying reproductive isolation (pollen and seed sterility) loci between two pairs of species, in order to draw inferences about the timing, accumulation, and underlying genetics of these loci across a genome.

### Methodological approach and assumptions

Implementing our cross-species tests of allelism and drawing general inferences from patterns of homologous and unique isolation loci relies on particular assumptions—about the expression of fertility phenotypes and our power to detect these loci in our mapping populations—that appear to be reasonable in our experiment (see Results). Several additional assumptions about the nature of evolutionary transitions underlying our QTL also appear to be reasonable, but will require knowledge of the underlying mutations for final confirmation. In particular, we assume that loci identified as homologous in our tests of allelism are not due to independent mutations in the same underlying gene. If recessive sterility effects are due to independent *change of function* mutations within the same locus, these mutations are expected to complement in our experiments. However, tests of allelism will not be able to differentiate homologous from non-homologous mutations in the same gene, if these different mutations have identical phenotypic effects. For sterility loci, this is most likely when the causal changes are loss-of-function mutations; if two such null mutations independently occurred in the same locus, a test of allelism would indicate the underlying alleles were the same (i.e. no fertility rescue would be observed in the IL_HP_ and/or IL_PH_ genotypes).

Several factors indicate that this scenario is unlikely to explain our data. Foremost, there are no known cases where hybrid sterility effects arise from interactions between loci with simple loss-of-function mutations. Theoretically, most models of hybrid incompatibility are based on sterility arising from dysfunctional interactions between loci that continue to have functional roles on their own native genetic background [1]–[3]. Empirically, of the molecular loci currently known to cause hybrid sterility, almost all are functional on their own genetic background but dysfunctional on a hybrid background (reviewed in [13], [14], [38], [39]). However, there are some cases where gene duplication followed by loss events has led to the placement of (still functional) homologous loci in different chromosomal locations in different species. This ‘gene movement’ based on divergent resolution of duplicates in alternative lineages can lead to the segregation of null genotypes in recombinant hybrid populations, e.g., [40]–[42] and reviewed in [14], as predicted by one model of incompatibility evolution [43], [44]. However, for gene movement or convergent loss-of-function mutations to explain our observations for ‘shared’ loci here, there must have been two independent gene movements, two independent loss-of-function mutations, or an independent movement and loss-of-function mutation, at the same locus in two different lineages (SH and SP). This is unlikely to be the case, certainly for both of the loci (*pf7.2*, *sss1.2.1*) that we infer share homologous sterility alleles.

Given this, and the large number of loci that could potentially contribute to dysfunctional sexual development in hybrids, it is more parsimonious to infer that co-localized sterility loci that show phenotypes consistent with allelism (i.e. that do not complement), are underpinned by the same mutation rather than two independent mutations in the same locus. This inference can be confirmed with further fine-mapping and ultimate identification of the underlying loci. Similar logic indicates that it is more parsimonious to infer that loci identified in only one species cross are due to a single lineage-specific change, rather than an initially shared change that has been secondarily lost or ameliorated in one lineage, especially as the accumulation of postzygotic sterility loci is generally considered be irreversible [45].

### Insights into the temporal accumulation of mutations underlying reproductive isolation

Differentiating earlier versus later evolving mutations involved in DMIs, by placing the evolution of these changes on specific evolutionary branches, provides both specific and general insight into the historical progression and evolutionary dynamics of speciation. The early-evolving mutations inferred here (*pf7.2* and *sss1.2.1*), for example, can be targeted for further genetic and functional characterization to examine the nature of changes that specifically accompanied the first stages of divergence among our species, and evaluate whether these differ from later-evolving substitutions. More generally, we can also use these empirical data to evaluate hypotheses about the genetic and evolutionary mechanisms underpinning divergence and speciation processes. At least three substantive conclusions about these processes emerge from our data:

#### Sterility effect sizes involving earlier- versus later-arising mutations do not differ

Although we have few ‘early’ mutations with which to make such a comparison, we find no evidence that DMIs involving earlier substitutions have larger effect sizes than those involving later substitutions (Table S5). Predictions about the expected effect sizes of early- versus late-arising mutations vary depending upon the assumptions made in different evolutionary models. For example, under Fisher's geometric model of adaptation via strong directional selection, effect sizes of the earliest mutations affecting fitness should be large; increasingly smaller effect mutations then accumulate as the population approaches its new phenotypic optimum [46]. However, there is no reason to expect that these conditions should apply to mutations underlying postzygotic isolation, whose general mechanistic connections to adaptation are unknown.

Other models specific to isolation-causing mutations have proposed that their phenotypic effect size might either increase with increasing divergence between lineages [45] or have no association with divergence [15]. Under an ‘infinitesimal’ model of sterility (i.e. sterility based on many genes of very minor effect), for example, it is argued that later substitutions have potentially larger phenotypic effects due to the aggregate effect of interacting with many changes that have occurred earlier [47]. Most current data indicate that DMIs are due to interactions among a limited (not infinitesimal) number of genes (reviewed in [13], [14], [38], [39]). However, if the likelihood of complex DMIs (those that involve interactions among >2 loci) increases as more substitutions accumulate between lineages [48], and if these complex DMIs have larger phenotypic effects than simple (pairwise) DMIs, then later evolving mutations might be expected to have larger average effect sizes. The relative effect size of pairwise versus more complex DMIs is currently unknown but, in principle, this is a potential source of systematic differences between early versus late substitution effects on isolation phenotypes.

In contrast, Orr [15] argues that the probability that a sterility-causing mutation has a particular effect size is mathematically independent of where it occurs in the order of substitutions between lineages ([15], page 1809). Although we do not yet know if any of our lineage-specific QTL are composed of more than one causal change (in which case, each independent late-arising mutation would likely have a smaller effect than we have estimated for ‘late’ loci), our data here suggest that there is little evidence of a systematic difference in effect size between DMIs that involve earlier versus later evolving mutations. These observations would therefore appear to support Orr's model [15] of the accumulation of isolating mutations.

#### Reproductive isolation mutations accumulate non-linearly over time, and involve complex interactions for male sterility

The fact that DMIs are caused by dysfunctional genetic interactions (epistasis) between alleles from at least two loci generates unique predictions about their evolutionary accumulation. In particular, Orr [15] showed that as each new mutation accumulates between lineages, the number of potential deleterious interactions into which it can enter—i.e., interactions with alleles at loci that have already experienced substitutions—increases combinatorially. Accordingly, the number of substitutions that affect reproductive isolation is predicted to increase at a pace greater than linearly with time—resulting in a ‘snowballing’ of hybrid incompatibility loci [15]. This predicted snowballing has previously been demonstrated for seed sterility loci in *Solanum*, by showing that the total number of sterility loci between species pairs increases significantly faster than linear with evolutionary divergence [30] (see also [31] for similar evidence in *Drosophila*).

Here we use a different approach, in which we assess the fit of our data to models that explicitly describe the distribution of isolating incompatibilities along a phylogeny, based on the different proportions of shared-to-unshared isolation loci these models predict [19]. This statistical phylogenetic approach also supports the inference that hybrid incompatibility loci accumulate non-linearly (snowball) with increasing divergence between lineages, for both pollen and seed sterility loci. In addition to differentiating linear from non-linear models, this more sophisticated analysis enabled us to examine alternative models of non-linear accumulation that specifically vary components of the underlying genetics of hybrid incompatibilities (Results, and see [19]); these models can therefore also provide insight into the genetics of DMIs that best fit our observations. We found that the distribution of shared-to-unshared loci is consistent with DMIs due to pairwise interactions for seed sterility, but with more complex (three-locus) interactions for pollen sterility.

**Figure 6:**
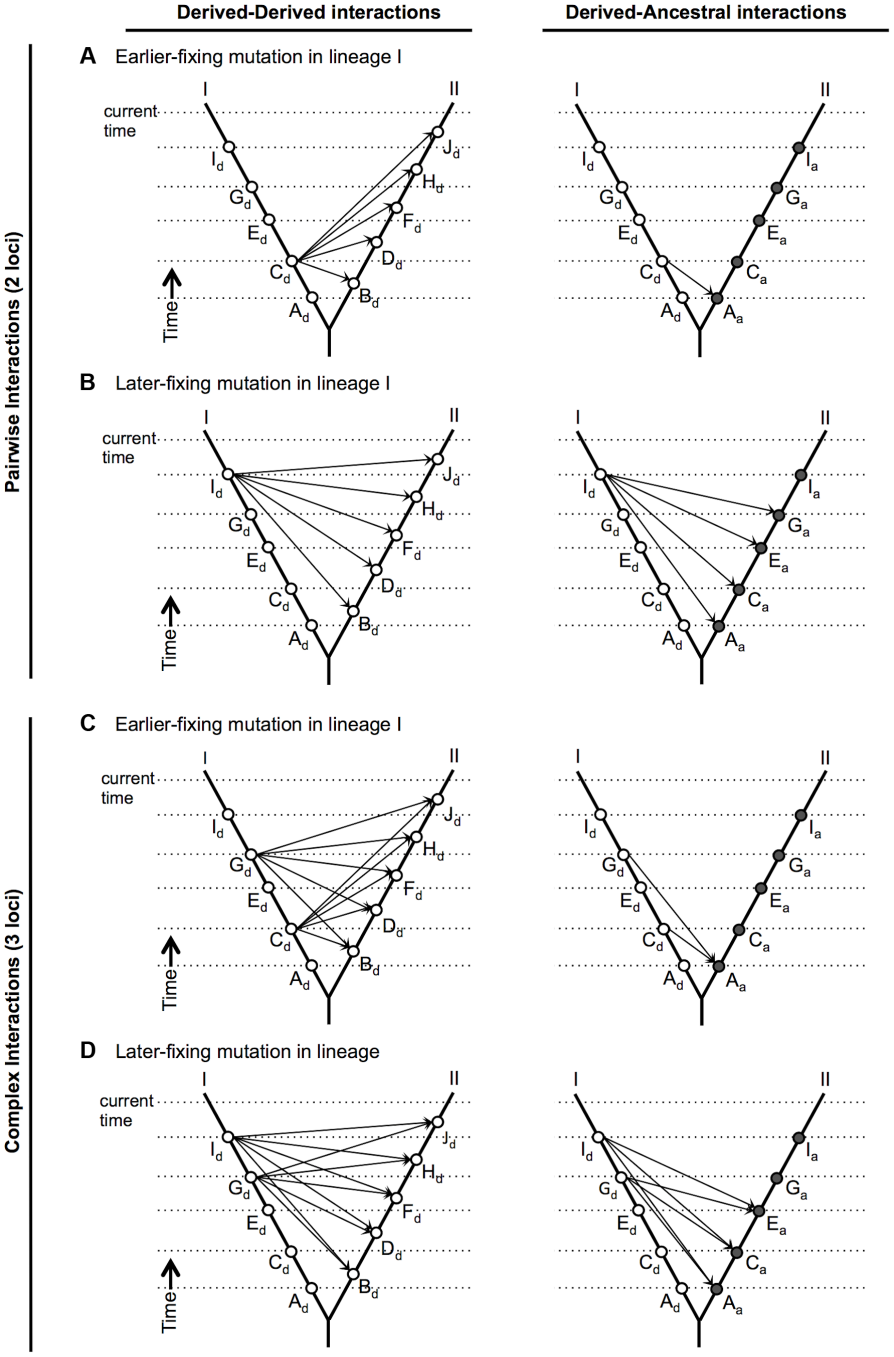
The number of potential deleterious interactions among derived alleles only (left panels), or including ancestral alleles (right panels), that involve a specific derived fixation in lineage I, under a combinatorial epistatic model of hybrid incompatibility accumulation [15]. The number of potential interactions is evaluated at the most recent (top) time point. Loci that have undergone substitutions in lineage I and II are labeled with unique letters. Derived alleles, denoted with subscript ‘d’, are depicted at their time of origin along each lineage using open circles. For alleles that are derived in lineage I, ancestral alleles at the homologous locus in lineage II are depicted with closed circles (and subscript ‘a’) in the right panels. Potential interactions are depicted with arrows between alleles for each relevant pair (panels A and B) or trio (panels C and D) of alleles. **A**) Pairwise interactions involving an early mutation in lineage I (at locus C). **B**) Pairwise interactions involving a later mutation in lineage I (at locus I). In general, for pairwise interactions, at time *t* the possible number of derived-derived interactions (left panels) involving the *i*th derived allele in one lineage is simply to total number of derived alleles in the other lineage, regardless of when the *i*th allele arose during divergence (A and B, left panels). In contrast, the possible number of derived-ancestral interactions involving the *i*th derived allele in one lineage is [*i*-1] (A and B, right panels). **C**) Complex (3-locus) interactions involving a lineage I fixation that occurred early in the history of divergence between I and II (at locus C). **D**) Complex (3-locus) interactions involving a lineage I fixation that occurred late in the history of divergence between I and II (at locus I). For both C) and D), the second derived mutation in lineage I is at locus G. In general, for complex (3-locus) interactions, except for the first derived allele, at time *t* the possible number of complex derived interactions involving the *i*th and *j*th derived alleles in one lineage is simply the total number of derived alleles in the other lineage, regardless of when the *i*th allele arose during divergence (C and D, left panels). In contrast, except for the first derived allele, the possible number of derived-derived-ancestral interactions involving the *i*th and *j*th derived alleles in one lineage is [*i*-1] or [*j*-1], whichever is smaller (C and D, right panels).

Satisfyingly, it can also be shown that this inference for pollen sterility is consistent with our observation that almost all (13/14) pollen sterility-associated mutations occurred on terminal branches (later in lineage divergence) rather than on the internal branch (earlier in divergence) of our three species phylogenetic tree. This observation implies that later-arising mutations are more likely to engage in deleterious interactions that cause pollen sterility, than are earlier-arising mutations. Importantly, the general snowball prediction that DMIs should become increasing prevalent over time [15] is agnostic about *when* the mutations involved in these interactions arise during the history of divergence. Indeed, it can be shown that for pairwise ‘derived-derived’ interactions between two species (interactions between loci that have both experienced a new mutation within their respective lineage), DMIs are no more likely to involve later-fixing mutations than earlier-fixing mutations (Figure 6A and B, left panels). Therefore, although the number of potential deleterious interactions snowballs over time, for pairwise derived-derived interactions, there is no expectation that a greater proportion of later-evolving mutations will be involved.

Nonetheless, there are at least two circumstances where later-evolving mutations have a higher likelihood of engaging in incompatible interactions (DMIs): when isolation is due to derived-ancestral pairwise interactions, and when it is based on complex (>pairwise) interactions. In the first case, derived-ancestral interactions [15] (and see Results) arise when multiple new mutations occur sequentially along one evolutionary branch (Figure 6). When the *i*th derived allele arises on a background of *i*-1 previously derived alleles, this allele has never been co-tested with the corresponding ancestral allele at each of these *i*-1 loci; as a consequence, it can potentially be incompatible with every ancestral allele at each of these loci in the other lineage (Figure 6A and B right panels). Because later-evolving alleles arise on increasingly more derived genetic backgrounds, they can be involved in increasingly more interactions with ancestral alleles in the other lineage. Accordingly, for this particular class of pairwise interactions only, the temporal order in which mutations accumulate between lineages will change their relative involvement in deleterious DMIs.

The same general inferences apply to DMIs that involve greater than pairwise (i.e., complex) interactions. (The only salient difference is that, because DMIs due to complex genetic interactions require the fixation of at least three allelic changes, the first derived fixation in any lineage does not participate in these interactions.) Complex DMIs due solely to interactions among derived mutations are no more likely to involve later versus earlier fixing mutations (Figures 6C and 6D, left panels). In comparison, as for pairwise interactions, complex DMIs involving ancestral alleles will also become more likely for later-fixing mutations (Figures 6C and 6D, right panels).

Although the specific effect of derived-ancestral and higher-order (complex) interactions on increasing the likelihood of engaging in DMIs over time was not emphasized in Orr's original treatment [15], it clearly emerges from this model when we consider the temporal accumulation of these loci. This is relevant to our findings because it provides an explanatory link between two of our results for pollen sterility loci: an apparently enriched involvement of later-arising mutations in DMIs, and support for a phylogenetic model whereby sterility includes complex DMIs. Although the first observation could be driven by pairwise derived-ancestral interactions, our phylogenetic model fits suggest that it is due to the influence of complex (>pairwise) genetic interactions on pollen sterility.

Together, these observations suggest that male sterility is especially influenced by a genetic architecture and/or evolutionary dynamics that enrich for the accumulation of serial, sequential substitutions that interact epistatically. Studies in both animals and plants provide some evidence that male sterility loci are both more likely to be strongly epistatic on previous mutations and/or that male sterility effects involve complex (>2 locus) interactions [49]–[54], (unpublished data). Such patterns of fixation could be consistent with antagonistic co-evolutionary processes, whereby compensatory counter-adaptations involve sequential fixations at epistatic loci [55]. In comparison, if interactions underlying female (ovule) sterility effects are generally less complex—involving only pairwise interactions—they will be more consistent with the expectations for derived-derived interactions. This is consistent with our phylogenetic model comparisons, which indicate that our seed data best fits isolation accumulation under the standard pairwise DM model.

#### Little evidence for repeated parallel involvement of the same loci in reproductive isolation

The extent to which specific reproductive isolation loci are shared among more than one species pair remains unknown. Although some studies of molecular evolution at known isolation genes have revealed patterns consistent with repeated evolutionary fixations at a single sterility locus [13], sometimes in more than one lineage, e.g. [26], it is not known whether this is a general characteristic of isolation loci. White et al. [29] recently found that most hybrid male sterility loci are not shared between mouse species crosses. Consistent with this observation, our data here—and similar data from other species pairs in this group (Text S1)—provide little evidence that the same genes or chromosomal regions have been consistently, independently, involved in the formation of postzygotic species barriers among these species. Although we have only 22 QTL and two species pairs to consider here, 20 of these isolation QTL are unshared between our pairs. Moreover, although we have argued that the two remaining shared isolation QTL are due to the same mutation event, even if these cases are counted as independent events, our data indicates a maximum ratio of 2∶20 for repeated∶unique loci involved in DMIs. This does not preclude that certain classes of genes, or developmental or functional processes, are more involved in the development/expression of hybrid sterility, but it does suggest that the specific loci involved reproductive isolation differ among speciation events.

These observations are quite different from several well-described cases of convergent adaptive phenotypic evolution—where evolution of the same phenotypic response is due to independent mutations at the same locus in different lineages, e.g. [56]–[58], and reviewed in [59]–[60]—and might suggest differences in the evolutionary forces and constraints predominantly shaping these two processes. Future identification of the molecular underpinnings of these loci can shed further light on this question.

### Conclusions

Tests of allelism can be used to assess whether isolation alleles detected between specific species are homologous with co-localized QTL detected in other crosses. Using this approach, in conjunction with data on loci that are unique to single species pairs, we infer the phylogenetic timing of mutations underlying all known reproductive isolation loci among three *Solanum* species. With these data, we determine which loci are associated with mutations that arose early versus late in lineage divergence between these species, compare properties of these loci, and observe that many of these isolation-associated mutations arose on more recent evolutionary branches. Using new phylogenetically informed analyses, we find clear support for the theoretical prediction that reproductive isolation loci accumulate non-linearly over evolutionary time. Moreover these analyses suggest that different sterility phenotypes having different underlying genetic architectures: seed sterility data are consistent with isolation due to pairwise epistasis, whereas pollen sterility data are consistent with more complex epistasis among loci that have experienced sequential fixations in one or both lineages. Therefore, while overall patterns of isolation accumulation fit a theoretical framework in which sterility is due to genetic interactions between alleles in diverging lineages, the complexity and nature of these interactions might differ depending upon the specific traits involved and the dynamics acting on these traits during their evolution.

## Materials and Methods

Introgression lines used in this experiment were derived from two introgression line populations of *S. habrochaites* (SH) or *S. pennellii* (SP) in a *S. lycopersicum* (SL) background. In each population, each introgression line (IL) contains a short, marker delimited, homozygous region from SH or SP, introgressed into an otherwise isogenic genetic background of the domesticated tomato SL [61]–[63]. The construction of these lines has been described extensively elsewhere [61]–[63], (and see Text S1). In previous studies with these populations [7], [27], we identified QTL associated with significantly reduced pollen and seed fertility, including QTL that appeared to be chromosomally co-localized in the two studies [27], where co-localization was inferred from overlap in the physical location of the heterospecific introgression. For *pf7.2*, to resolve the chromosomal position of each introgression boundary more finely than in the original mapping studies, and therefore clarify the degree of overlap between SH and SP introgressions at this QTL, we performed additional genotyping of IL_PP_ and IL_HH_ lines (Text S1 and Table S9).

At each QTL location—*pf7.2*, *sss1.2*, and *sss2.1*—the carrier ILs (IL_HH_ and IL_PP_) were crossed in both directions to generate IL_HP_ and IL_PH_ individuals that are isogenic SL except in the region of the QTL where they carry alleles from the two alternative donor species, as described in the Results (Figure 1). For completeness, we also assessed complementation at an additional pair of pollen sterility QTL, located on the long arm of chromosome 9, that were closely adjacent but not overlapping in the two mapping studies (Table S1, Figure S3, Text S1 and Text S2).

For each QTL pairing, we evaluated fertility in the following genotypic classes: *S. lycopersicum* (SL), IL_PP_, IL_HH_, IL_HP_, and IL_PH_ (Figure 1). Note that only one IL combination (for *pf 7.2*) successfully produced IL_PH_ seeds (Text S2), so this was the only locus for which we evaluated fertility in IL_PH_ genotypes. Sample sizes for each genotypic class were at least 25 (i.e. 25 replicate plants per class). Experimental individuals were cultivated under controlled greenhouse conditions as previously described [7] (Text S1). Fertility was evaluated as for both previous QTL mapping experiments with these lines [7], [27] (Text S1). Pollen fertility (PF) was estimated as the proportion of fertile pollen/per flower, averaged from subsamples of lactophenol aniline blue-stained pollen from three flowers per plant. Seed fertility was estimated as total seeds per fruit after controlled hand-pollination (selfed seed set; SSS), averaged from four fruits per individual.

### Phylogenetic tree and branch length estimation

Estimated branch lengths for our three species tree were drawn from a whole clade (13 species, plus one outgroup) ultrametric tree generated using RAxML Pthreads 7.0.0 [36], based on 18 previously published loci [37] (Figure S2). Results reported for RAxML ultrametric branch lengths (Tables S6 and S7) are indistinguishable from analyses using the alternative Bayesian tree-building method MrBayes [37]; in particular, median branch length values in the MrBayes data are identical to mean branch length estimates from the RAxML analysis. Variability in estimation of the phylogeny was incorporated in two ways. For our assessment of whether isolation-causing mutations were linearly proportionate to branch lengths, standard deviations for branch lengths on the RAxML tree were obtained from 100 bootstrap replicates, using the topology from the best RAxML partitioned tree (Text S1). Branch length standard deviation estimates were used to evaluate the effect of substantial estimation error on our inferences (Table S6 and Text S2). For our comparison of phylogenetic models of incompatibility accumulation, we estimated branch lengths using trees drawn from the posterior distribution of our MrBayes analysis [37]. This posterior was represented by sampling every 100 generations from a Markov chain running for 10,000,000 generations, discarding the first 25% of trees as burn-in. Since the mathematical description of incompatibility accumulation assumes substitutions accumulate at a constant rate between different branches, we used a penalized-likelihood method, as implemented in *R*/*ape*, to enforce a molecular clock on the branch lengths drawn from the posterior.

### Analysis

For each fertility measure (SSS or PF) within each QTL pairing, we ran a nested ANOVA with genotype (4 or 5 levels, depending upon whether the IL_PH_ genotype could be made—Text S1), and maternal parent nested within genotype. In every case we detected a significant effect of genotype (Results, Table S2); in no case did we detect a significant effect of maternal parent (Table S2). We used least-squares means (LSMeans) contrasts (Tukey HSD tests of all pairwise contrasts) to assess the fertility of IL_PH_ and IL_HP_ genotypes relative to fertility of the homozygous IL parents (IL_PP_, IL_HH_) and the recurrent SL parent. For completeness, we assessed patterns of fertility for both pollen and seed fertility phenotypes, regardless of whether the original QTL was identified for PF or SSS; only results for cases where we have an expectation of potential homology are reported in the main text. Because hybrid sterility generally acts partially or fully recessively [27], a significant increase in fertility in IL_HP_ and/or IL_PH_ genotypes, in comparison to one or both parental ILs, is consistent with rescue/complementation of the sterility phenotype, and therefore the inference that underlying loci are not homologous.

Bootstrap analyses to compare early versus late effect sizes, and binomial resampling to evaluate the pattern of accumulation of mutations over time, were performed in R (R Development Core 2009) using scripts as described in [64] (page 385 and 365, respectively). Analyses of phylogenetically explicit models of incompatibility accumulation were performed using custom Python scripts as described in [19]. Prior simulations suggest there is low power to distinguish alternative non-linear models in datasets with few (<20) isolation QTL [19]; these comparisons are particularly prone to Type 2 error (false negatives, or incorrect acceptance of the simpler model). Simulations parameterized with data from this study similarly suggest that our comparisons have a higher propensity of returning false negatives when comparing simpler to more complex models (Results); nonetheless, these simulations also suggest greatest support for non-linear models of sterility accumulation. Therefore, although we have relatively few loci to fit such models here, our inference that sterility data support more complex (non-linear) models is robust.

## Supporting Information

Figure S1 Inferring the evolutionary timing of reproductive isolation loci from comparative QTL mapping. A) A schematic of the genomic location of isolation QTL acting between species pairs *sp1×sp2* (red, light bars) and *sp1×sp3* (blue, dark bars). Roman numerals indicate chromosomes 1 to 4. Each QTL is numbered (in italics). Two QTL (*3*, *5*) are detected in both species pairs; 5 QTL are unique to a single species cross. B) A rooted phylogeny showing relationships among species, with individual branches labeled. The QTL detected in only one species cross must be due to changes that occurred on an evolutionary branch that is unique to that species cross; therefore QTL unique to pair *sp1×sp2* (i.e., *2*, *7*) arose on branch *c*; QTL unique to pair *sp1×sp3* (i.e., *1*, *4*, *6*) arose on branch *b*. Shared QTL (*3*, *5*) must have arisen on branches that are shared among all three species (branch *a* or *d*). (Modified from [20]).
(TIFF)

Figure S2 Phylogenetic relationships among 13 *Solanum* species, based on 18 unlinked loci, and generated in RAxML as described in [37]. Branch lengths are given above each branch; standard deviations for the focal branches are given below. The three species in the present study are boxed (unbroken line): SL = *S. lycopersicum*; SH = *S. habrochaites*; SP = *S. pennellii*. Two additional species for which there are also mapping data are boxed (broken line): SC = *S. chilense*; SM = *S. pimpinellifolium*. LCA = last common ancestor of all 5 boxed species; MRCA = most recent common ancestor of SL, SM, SC. Branches *b, c, d* are the same as those depicted in Figure 5. Branch *f* denotes the branch shared by SL, SM, and SC.
(TIFF)

Figure S3 Test of fertility in adjacent pollen sterility loci at *pf9.1*. A) Chromosomal location (shaded) of SP and SH introgressions represented in IL_PP_ and IL_HH_ lines, respectively, used in cross-species tests. Marker IDs (solgenomics.org) are shown to the right (SP) or left (SH) of chromosome 9. B) Pollen fertility (percent fertile pollen) in 5 genotypes (SL = *S. lycopersicum*; IL_HH_ = introgression line with homozygous SH alleles; IL_PP_ = introgression line with homozygous SP alleles; IL_HP_ = heterointrogression line (from IL_HH_ maternal parent); IL_PH_ = heterointrogression line (from IL_PP_ maternal parent)).
(TIFF)

Table S1 QTL for pollen (PF) and seed (SSS) fertility in two previous mapping experiments [7], [27]. QTL are listed in linear order along chromosomes (1 through 12); loci that are physically co-localized in the two experiments are identified in columns 6 and 11. Note that *pf9.1* is not co-localized; the relevant introgressions are adjacent but not overlapping. delta% describes the percentage phenotypic change from the SL genotype. * *sss* loci that were not statistically independent of associated pollen sterility. ** for consistency between the two mapping studies, this locus is re-labelled *pf7.2* in the current study. ***sss7.1 is co-localized with, and statistically dependent upon, *pf7.2*.
(DOCX)

Table S2 Nested analyses of variance of pollen and seed fertility in introgression line genotypes at four reproductive isolation QTL. Effects are genotype and maternal family nested within genotype; significant effects are in bold. Least squares means contrasts are based on Tukey's Honest Significant Difference (HSD) (i.e. corrected for multiple tests). Genotypes are defined in the main text.
(DOCX)

Table S3 Effect sizes of sterility QTL (DMIs) associated with mutations that are inferred to have arisen earlier (shared) versus later (lineage-specific) during lineage divergence. * effect size estimated from previous mapping experiments. ** effect size estimated from this experiment. ł estimated individual effect, apart from adjacent QTL, in this experiment.
(DOCX)

Table S4 Multivariate analyses of variance of seed fertility in introgression line genotypes at four reproductive isolation QTL, taking into account pollen fertility in the same genotypes. Effects are genotype, maternal family nested within genotype, and pollen fertility (PF); significant effects are in bold. Least squares means contrasts are based on Tukey's Honest Significant Difference (HSD) (i.e. corrected for multiple tests).
(DOCX)

Table S5 Effect sizes of DMIs associated with earlier evolving versus later evolving sterility-causing mutations. delta% describes the proportional reduction in fertility in comparison to the fully fertile recurrent parent (SL), as in [7], [27]. Bootstrap analysis was performed on delta% values because these account for differences in average fertility of the SL genotype between different experiments (Text S1). Analysis results are given for effect size comparisons on two possible cuts of the dataset for each of our fertility traits: the first preferentially uses effect size estimates from the original analyses (where possible) and the second preferentially uses estimates from the analyses here (where possible) (Text S2).
(DOCX)

Table S6 Tests of the distribution of observed QTL on early versus late branches, contingent upon observed branch lengths, using χ^2^ goodness-of-fit tests. Two different models of incompatibility accumulation are evaluated: ‘linear’ and ‘exponential’ models. Two cases that evaluate uncertainty in branch length are also included (Text S2). ‘Extrapolated loci’ indicates the case in which we assume that 1/3 of our lineage-specific QTL are underpinned by two mutations. Significant comparisons are in bold.
(DOCX)

Table S7 Tests of the distribution of observed QTL on early versus late branches, contingent upon observed branch lengths (‘RAxML’), using binomial resampling. For comparison, expectations and results from the same tests—but assuming equal branch lengths on branches *b, c*, and *d* in the three species phylogeny (‘Equal’)–are also shown. Significant comparisons are in bold. For each, expected p_early_ = (1-p_late_). All bootstrap simulations performed with N = 1000. ‘Extrapolated # loci’ indicates the case in which we assume that 1/3 of our lineage-specific QTL are underpinned by two mutations. * rounded to nearest whole number.
(DOCX)

Table S8 Comparative QTL results for the original mapping studies (‘previous threshold’) and under two more permissive statistical thresholds for detecting QTL in each mapping population, for A) pollen fertility and B) seed fertility QTL. MP = moderately permissive threshold; HP = highly permissive threshold (Text S1). QTL are listed in linear order along chromosomes (1 through 12); detected loci that are physically co-localized with a QTL detected in the other species cross are identified in the final column for each population. New QTL detected only under one or both more permissive statistical thresholds are shown in bold; note that in most cases, new ILs found to be statistically significant under more permissive thresholds coincided with a chromosomal location where a significant QTL had already been detected in an overlapping IL from the same population under a more stringent statistical cut-off. * sss loci that were not statistically independent of associated pollen sterility effects. **for consistency between the two mapping studies, this locus is re-labeled *pf7.2* in the current study. ***sss7.1 is co-localized with, and statistically dependent upon, *pf7.2*. ł one line supports two previously identified QTL. łł unknown which of these are independent of pollen sterility.
(DOCX)

Table S9 Primer pairs used for additional genotyping on chromosome 7. For marker TG61, primer sequences on SolGenomics were used.
(DOCX)

Text S1 Supporting information for materials and methods, including details of plant handling and cultivation, fertility evaluation, ancillary marker genotyping, phylogenetic analysis, and assessment of QTL detection under more permissive statistical thresholds.
(DOCX)

Text S2 Supporting information for results, including pollen/seed multivariate analyses, QTL effect size comparisons, inferences about the phylogenetic placement of loci, evidence for the unequal distribution of sterility-causing mutations over time, and additional sterility phenotypes not associated with tests of allelism.
(DOCX)
