# Supplementary figures and images for: Interspecific Tests of Allelism Reveal the Evolutionary Timing and Pattern of Accumulation of Reproductive Isolation Mutations
Source: PLoS Genet. 2014 Sep 11;10(9):e1004623. doi: 10.1371/journal.pgen.1004623 (PMC4161300; doi:10.1371/journal.pgen.1004623)

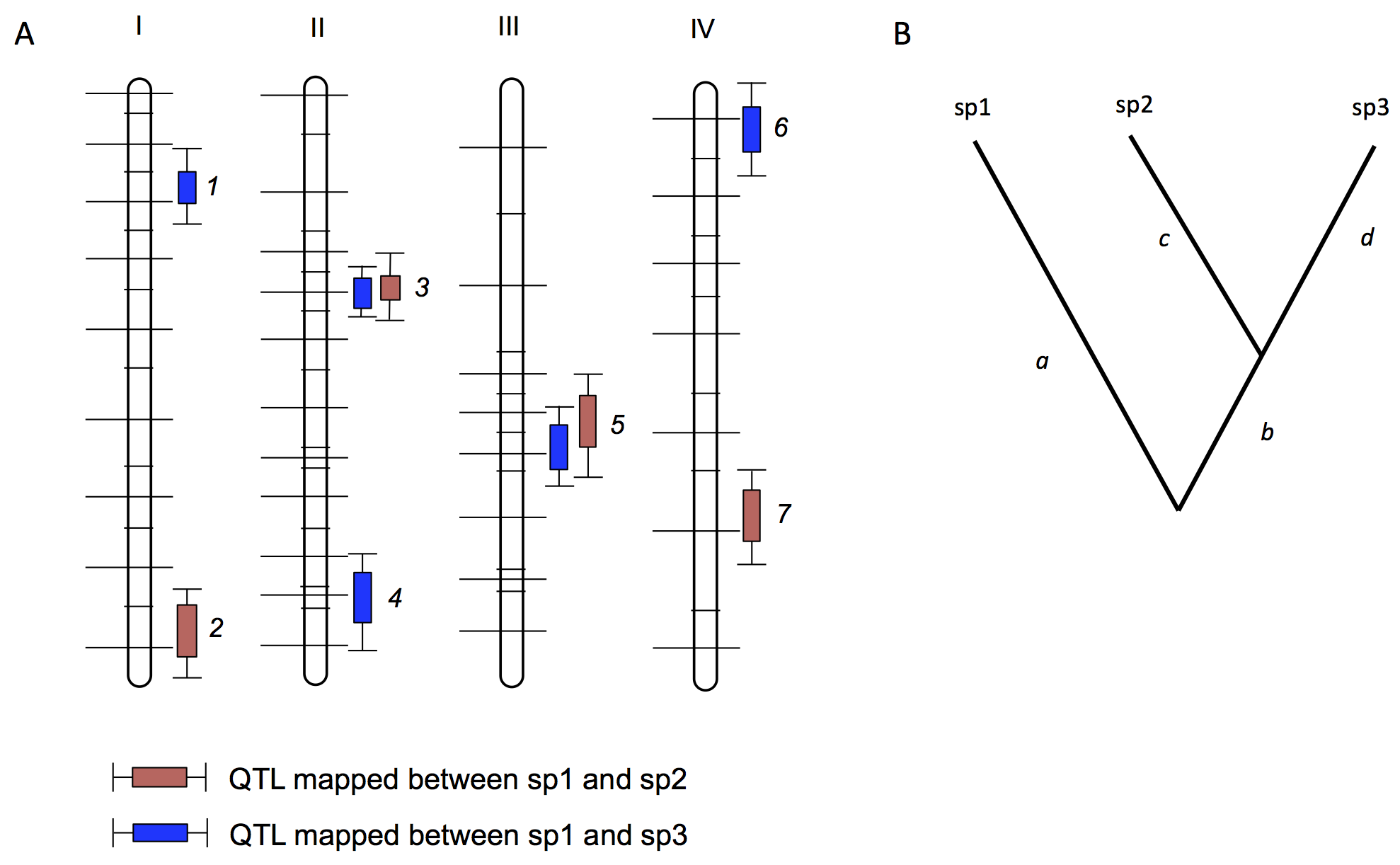

Supplement: Figure S1 — Inferring the evolutionary timing of reproductive isolation loci from comparative QTL mapping. A) A schematic of the genomic location of isolation QTL acting between species pairs sp1×sp2 (red, light bars) and sp1×sp3 (blue, dark bars). Roman numerals indicate chromosomes 1 to 4. Each QTL is numbered (in italics). Two QTL (3, 5) are detected in both species pairs; 5 QTL are unique to a single species cross. B) A rooted phylogeny showing relationships among species, with individual branches labeled. The QTL detected in only one species cross must be due to changes that occurred on an evolutionary branch that is unique to that species cross; therefore QTL unique to pair sp1×sp2 (i.e., 2, 7) arose on branch c; QTL unique to pair sp1×sp3 (i.e., 1, 4, 6) arose on branch b. Shared QTL (3, 5) must have arisen on branches that are shared among all three species (branch a or d). (Modified from [20]). (TIFF) [file pgen.1004623.s001.tiff]

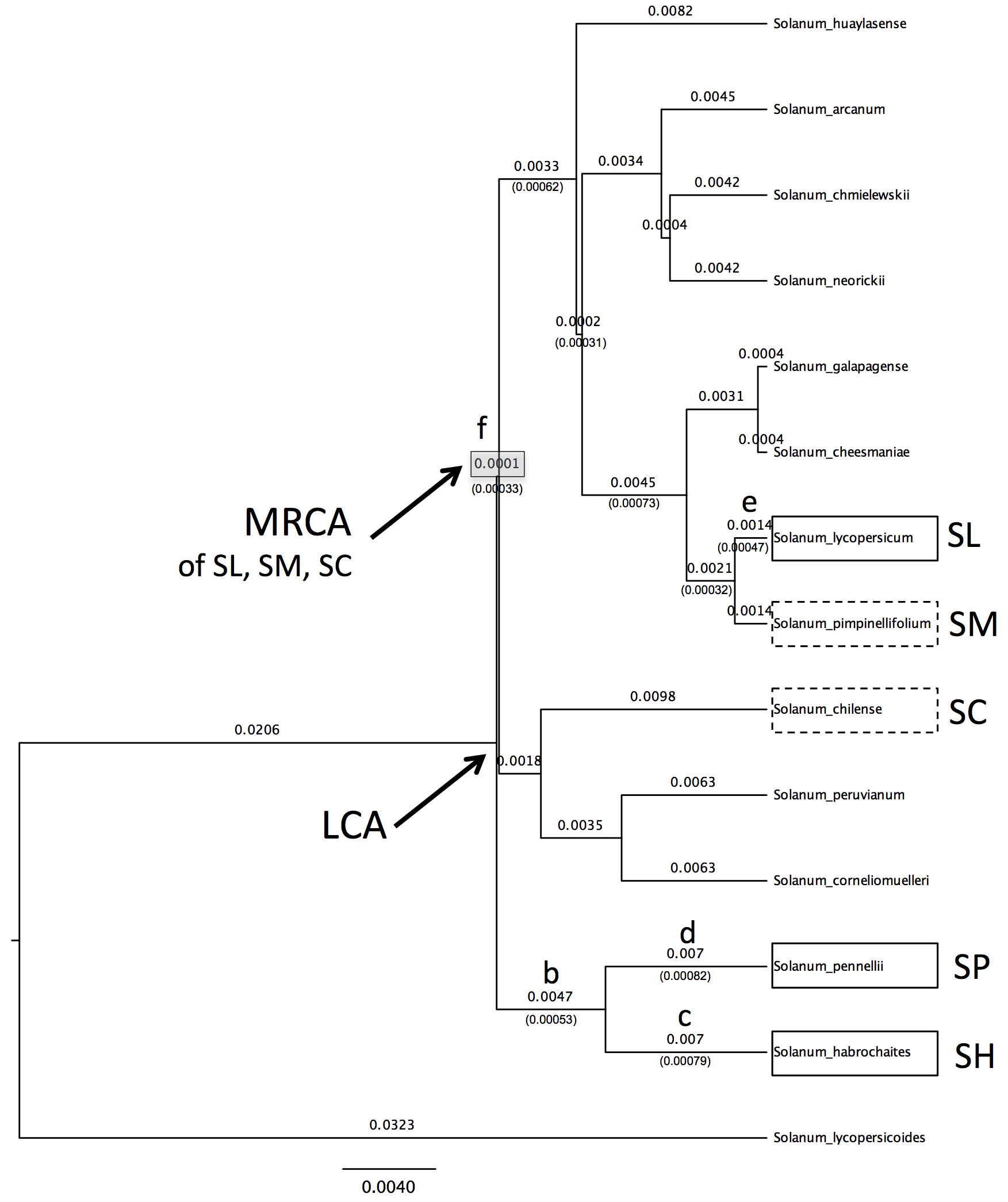

Supplement: Figure S2 — Phylogenetic relationships among 13 Solanum species, based on 18 unlinked loci, and generated in RAxML as described in [37]. Branch lengths are given above each branch; standard deviations for the focal branches are given below. The three species in the present study are boxed (unbroken line): SL = S. lycopersicum; SH = S. habrochaites; SP = S. pennellii. Two additional species for which there are also mapping data are boxed (broken line): SC = S. chilense; SM = S. pimpinellifolium. LCA = last common ancestor of all 5 boxed species; MRCA = most recent common ancestor of SL, SM, SC. Branches b, c, d are the same as those depicted in Figure 5. Branch f denotes the branch shared by SL, SM, and SC. (TIFF) [file pgen.1004623.s002.tiff]

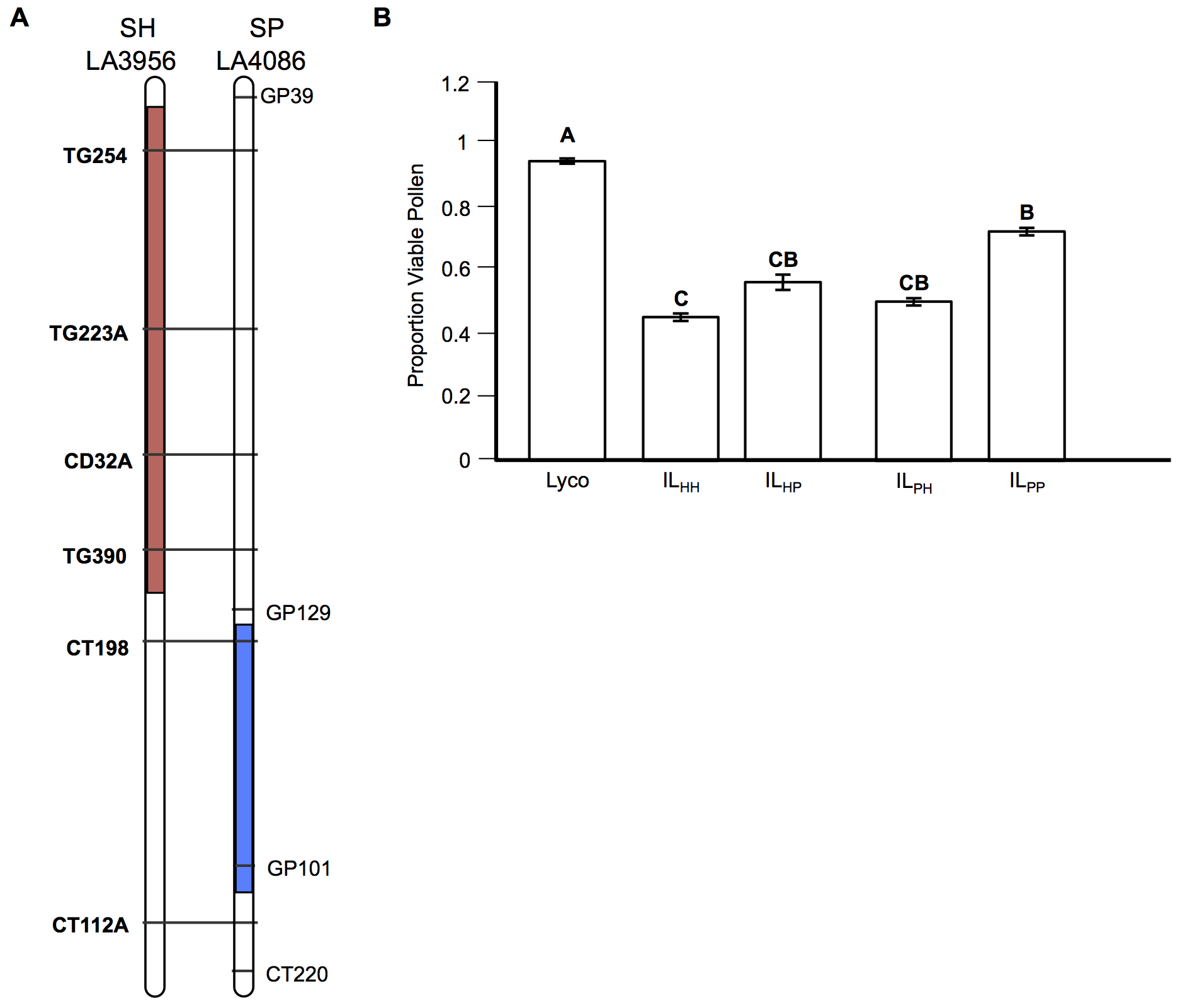

Supplement: Figure S3 — Test of fertility in adjacent pollen sterility loci at pf9.1. A) Chromosomal location (shaded) of SP and SH introgressions represented in ILPP and ILHH lines, respectively, used in cross-species tests. Marker IDs (solgenomics.org) are shown to the right (SP) or left (SH) of chromosome 9. B) Pollen fertility (percent fertile pollen) in 5 genotypes (SL = S. lycopersicum; ILHH = introgression line with homozygous SH alleles; ILPP = introgression line with homozygous SP alleles; ILHP = heterointrogression line (from ILHH maternal parent); ILPH = heterointrogression line (from ILPP maternal parent)). (TIFF) [file pgen.1004623.s003.tiff]
