# Supplementary material for: Interspecific Tests of Allelism Reveal the Evolutionary Timing and Pattern of Accumulation of Reproductive Isolation Mutations
Source: PLoS Genet. 2014 Sep 11;10(9):e1004623. doi: 10.1371/journal.pgen.1004623 (PMC4161300; doi:10.1371/journal.pgen.1004623)
Supplement: Table S1 — QTL for pollen (PF) and seed (SSS) fertility in two previous mapping experiments [7], [27]. QTL are listed in linear order along chromosomes (1 through 12); loci that are physically co-localized in the two experiments are identified in columns 6 and 11. Note that pf9.1 is not co-localized; the relevant introgressions are adjacent but not overlapping. delta% describes the percentage phenotypic change from the SL genotype. * sss loci that were not statistically independent of associated pollen sterility. ** for consistency between the two mapping studies, this locus is re-labelled pf7.2 in the current study. ***sss7.1 is co-localized with, and statistically dependent upon, pf7.2. (DOCX) [file pgen.1004623.s004.docx]

**Table S1**: QTL for pollen (PF) and seed (SSS) fertility in two previous mapping experiments (Moyle and Graham (2005), and Moyle and Nakazato (2008)). QTL are listed in linear order along chromosomes (1 through 12); loci that are physically co-localized in the two experiments are identified in columns 6 and 11. Note that *pf9.1* is not co-localized; the relevant introgressions are adjacent but not overlapping. delta% describes the percentage phenotypic change from the SL genotype

* *sss* loci that were not statistically independent of associated pollen sterility effects

** for consistency between the two mapping studies, this locus is re-labelled *pf7.2* in the current study

****sss7.1* is colocalized with, and statistically dependent upon, *pf7.2*

|  | **Population 1: SL x SH** | | |  |  | **Population 2: SL x SP** | | | |  |  | | |  |  |
| --- | --- | --- | --- | --- | --- | --- | --- | --- | --- | --- | --- | --- | --- | --- | --- |
| **Trait** | **Csome** | **QTL name** | **Mean phenotype** | **Delta%** | **Colocalized (QTL in other population)** | **Csome** | **QTL name** | | **Mean phenotype** | **Delta%** | **Colocalized**  **(QTL in other population)** | | |  |  |
| PF | 1 | pf1.1 | 0.56 | -33.3 | N | 1 | pf1.1 | | 0.57 | -41.8 | N | |  |  |  |
| PF | 2 | pf2.1 | 0.49 | -41.7 | N | 3 | pf3.1 | | 0.63 | -27.1 | N | |  |  |  |
| PF | 4 | pf4.1 | 0.58 | -31.0 | N | 4 | pf4.1 | | 0.58 | -33.4 | N | |  |  |  |
| PF | 6 | pf6.1 | 0.45 | -46.4 | N | 7 | pf7.1** | | 0.54 | -37.7 | Y (pf7.2) | |  |  |  |
| PF | 7 | pf7.1 | 0.59 | -29.8 | N | 8 | pf8.1 | | 0.53 | -52.4 | N | |  |  |  |
| PF | 7 | pf7.2 | 0.51 | -39.3 | Y (pf7.1) | 9 | pf9.1 | | 0.66 | -31.7 | N | |  |  |  |
| PF | 9 | pf9.1 | 0.58 | -31.0 | N | 11 | pf11.1 | | 0.67 | -26.9 | N | |  |  |  |
| PF | 10 | pf10.1 | 0.49 | -41.7 | N |  | - | |  |  |  | |  |  |  |
|  |  |  |  |  |  |  |  | |  |  |  | |  |  |  |
| SSS | 2 | sss1.2 | 6.07 | -89.1 | Y (sss1.2) | 1 | sss1.1 | | 20.97 | -70.7 | N | |  |  |  |
| SSS | 4 | sss4.1 | 20.10 | -63.8 | N | 1 | sss1.2 | | 12.20 | -82.9 | Y (sss1.2) | |  |  |  |
| SSS | 5 | sss5.1 | 9.00 | -83.8 | N | 2 | sss2.1 | | 26.82 | -62.5 | Y (sss2.1*) | |  |  |  |
| SSS | 8 | sss8.1 | 10.40 | -81.3 | N | 4 | sss4.1 | | 21.00 | -70.6 | N | |  |  |  |
| SSS | 1 | sss1.1* | 9.19 | -83.4 | N | 7 | sss7.1* | | 27.00 | -62.3 | Y (sss7.1*)*** | | |  |  |
| SSS | 3 | sss2.1* | 6.42 | -74.2 | Y (sss2.1) | 8 | sss8.1* | | 30.10 | -57.9 | N | |  |  |  |
| SSS | 4 | sss4.2* | 11.40 | -79.5 | N | 9 | sss9.1* | | 33.10 | -53.7 | N | |  |  |  |
| SSS | 7 | sss7.1* | 14.00 | -74.8 | Y (sss7.1*)*** | | |  |  |  | |  | | |  |
