# Supplementary material for: Interspecific Tests of Allelism Reveal the Evolutionary Timing and Pattern of Accumulation of Reproductive Isolation Mutations
Source: PLoS Genet. 2014 Sep 11;10(9):e1004623. doi: 10.1371/journal.pgen.1004623 (PMC4161300; doi:10.1371/journal.pgen.1004623)
Supplement: Table S2 — Nested analyses of variance of pollen and seed fertility in introgression line genotypes at four reproductive isolation QTL. Effects are genotype and maternal family nested within genotype; significant effects are in bold. Least squares means contrasts are based on Tukey's Honest Significant Difference (HSD) (i.e. corrected for multiple tests). Genotypes are defined in the main text. (DOCX) [file pgen.1004623.s005.docx]

**Table S2**: Nested analyses of variance of pollen and seed fertility in introgression line genotypes at four reproductive isolation QTL. Effects are genotype and maternal family nested within genotype; significant effects are in bold. Least squares means contrasts are based on Tukey's Honest Significant Difference (HSD) (i.e. corrected for multiple tests). Genotypes are defined in the main text.

|  |  | **ANOVA Whole Model** |  |  |  |  |  | **Effect Tests** |  |  |  |  | **Contrasts** |  |  |  |
| --- | --- | --- | --- | --- | --- | --- | --- | --- | --- | --- | --- | --- | --- | --- | --- | --- |
| **QTL** | **Trait** | **Source** | **DF** | **SS** | **MS** | **F** | **P** | **Source** | **DF** | **SS** | **F** | **P** | **Genotype** | **Least Squares Mean** | **Std Error** | **HSD** |
| *sss1.2* | seed | Model | 7 | 33382.006 | 4768.86 | 34.68 | <0.0001 | Genotype | 3 | 32603.813 | 79.0337 | **<0.0001** | SL | 60.825521 | 2.3176477 | A |
|  |  | Error | 80 | 11000.813 | 137.51 |  |  | Geno(MatFam) | 4 | 1241.922 | 2.2579 | 0.0701 | IL_PP | 10.41 | 2.3452945 | C |
|  |  |  |  |  |  |  |  |  |  |  |  |  | IL_HH | 38.132353 | 2.8440874 | B |
|  |  |  |  |  |  |  |  |  |  |  |  |  | IL_HP | 26.664062 | 6.0332204 | B |
|  |  |  |  |  |  |  |  |  |  |  |  |  | IL_PH | - | - |  |
| *sss1.2* | pollen | Model | 7 | 0.1644878 | 0.023498 | 2.0726 | 0.0557 | Genotype | 3 | 0.08005183 | 2.3536 | 0.0781 | SL | 1.3341926 | 0.02104467 | A |
|  |  | Error | 82 | 0.9296896 | 0.011338 |  |  | Geno(MatFam) | 4 | 0.05605942 | 1.2361 | 0.3021 | IL_PP | 1.2599248 | 0.0204918 | A |
|  |  |  |  |  |  |  |  |  |  |  |  |  | IL_HH | 1.3178813 | 0.02582484 | A |
|  |  |  |  |  |  |  |  |  |  |  |  |  | IL_HP | 1.3215814 | 0.05478275 | A |
|  |  |  |  |  |  |  |  |  |  |  |  |  | IL_PH | - | - |  |
| *sss2.1* | seed | Model | 6 | 32510.296 | 5418.38 | 12.8126 | <0.0001 | Genotype | 3 | 31668.67 | 24.9618 | **<0.0001** | SL | 60.825521 | 4.0643988 | A |
|  |  | Error | 80 | 33831.604 | 422.9 |  |  | Geno(MatFam) | 4 | 1226.922 | 0.9671 | 0.4125 | IL_PP | 11.076923 | 5.7035418 | B |
|  |  |  |  |  |  |  |  |  |  |  |  |  | IL_HH | 64.885802 | 3.957623 | A |
|  |  |  |  |  |  |  |  |  |  |  |  |  | IL_HP | 67.934211 | 4.7177997 | A |
|  |  |  |  |  |  |  |  |  |  |  |  |  | IL_PH | - | - |  |
| *sss2.1* | pollen | Model | 6 | 0.8013757 | 0.133563 | 3.1812 | 0.0075 | Genotype | 3 | 0.61973551 | 4.9202 | **0.0035** | SL | 1.3341926 | 0.04026889 | A |
|  |  | Error | 80 | 3.358839 | 0.041985 |  |  | Geno(MatFam) | 3 | 0.05602124 | 0.4498 | 0.7217 | IL_PP | 1.2105852 | 0.05683003 | AB |
|  |  |  |  |  |  |  |  |  |  |  |  |  | IL_HH | 1.1484399 | 0.03921098 | B |
|  |  |  |  |  |  |  |  |  |  |  |  |  | IL_HP | 1.1296843 | 0.04674259 | B |
|  |  |  |  |  |  |  |  |  |  |  |  |  | IL_PH | - | - |  |
| *pf7.2* | seed | Model | 8 | 2982.582 | 372.823 | 8.5357 | <0.0001 | Genotype | 4 | 2818.9953 | 16.135 | **<0.0001** | SL | 20.29256 | 1.1514975 | A |
|  |  | Error | 189 | 8255.187 | 43.768 |  |  | Geno(MatFam) | 4 | 128.8679 | 0.7376 | 0.5674 | IL_PP | 9.625316 | 0.975359 | B |
|  |  |  |  |  |  |  |  |  |  |  |  |  | IL_HH | 9.847067 | 0.9753953 | B |
|  |  |  |  |  |  |  |  |  |  |  |  |  | IL_HP | 10.71875 | 1.1683086 | B |
|  |  |  |  |  |  |  |  |  |  |  |  |  | IL_PH | 11.063725 | 1.1334258 | B |
| *pf7.2* | pollen | Model | 8 | 0.7737302 | 0.096716 | 3.3232 | 0.0014 | Genotype | 4 | 0.70154983 | 6.0263 | **0.0001** | SL | 1.240965 | 0.02973719 | A |
|  |  | Error | 189 | 5.5005572 | 0.029103 |  |  | Geno(MatFam) | 4 | 0.07755621 | 0.6662 | 0.6162 | IL_PP | 1.1146628 | 0.02517702 | B |
|  |  |  |  |  |  |  |  |  |  |  |  |  | IL_HH | 1.0635793 | 0.02517796 | B |
|  |  |  |  |  |  |  |  |  |  |  |  |  | IL_HP | 1.0901031 | 0.03017133 | B |
|  |  |  |  |  |  |  |  |  |  |  |  |  | IL_PH | 1.0816722 | 0.02927049 | B |
| *pf9.1* | seed | Model | 7 | 66975.16 | 9567.88 | 8.9139 | <0.0001 | Genotype | 4 | 63063.229 | 14.6882 | **<0.0001** | SL | 60.82552 | 6.475218 | B |
|  |  | Error | 86 | 92309.67 | 1073.37 |  |  | Geno(MatFam) | 3 | 1226.922 | 0.381 | 0.7669 | IL_PP | 41.95556 | 8.4591893 | B |
|  |  |  |  |  |  |  |  |  |  |  |  |  | IL_HH | 108.58333 | 7.3258728 | A |
|  |  |  |  |  |  |  |  |  |  |  |  |  | IL_HP | 111.35 | 8.4591893 | A |
|  |  |  |  |  |  |  |  |  |  |  |  |  | IL_PH | 71.65625 | 8.1905748 | B |
| *pf9.1* | pollen | Model | 7 | 6.106896 | 0.872414 | 8.7103 | <0.0001 | Genotype | 4 | 5.4754278 | 13.6669 | **<0.0001** | SL | 1.3341926 | 0.06254951 | A |
|  |  | Error | 86 | 8.613629 | 0.100158 |  |  | Geno(MatFam) | 3 | 0.0560212 | 0.1864 | 0.9054 | IL_PP | 1.0039149 | 0.08171433 | B |
|  |  |  |  |  |  |  |  |  |  |  |  |  | IL_HH | 0.735407 | 0.07076669 | B |
|  |  |  |  |  |  |  |  |  |  |  |  |  | IL_HP | 0.8121191 | 0.08171433 | B |
|  |  |  |  |  |  |  |  |  |  |  |  |  | IL_PH | 0.7781205 | 0.07911956 | B |
