# Supplementary material for: Interspecific Tests of Allelism Reveal the Evolutionary Timing and Pattern of Accumulation of Reproductive Isolation Mutations
Source: PLoS Genet. 2014 Sep 11;10(9):e1004623. doi: 10.1371/journal.pgen.1004623 (PMC4161300; doi:10.1371/journal.pgen.1004623)
Supplement: Table S3 — Effect sizes of sterility QTL (DMIs) associated with mutations that are inferred to have arisen earlier (shared) versus later (lineage-specific) during lineage divergence. * effect size estimated from previous mapping experiments. ** effect size estimated from this experiment. ł estimated individual effect, apart from adjacent QTL, in this experiment. (DOCX) [file pgen.1004623.s006.docx]

**Table S3**: Effect sizes of sterility QTL (DMIs) associated with sterility-causing mutations that are inferred to have arisen earlier (shared) versus later (lineage-specific) during lineage divergence.

* effect size estimated from previous mapping experiments

** effect size estimated from this experiment

ł estimated individual effect, apart from adjacent QTL, in this experiment

| **Class of Locus** | Lineage | **QTL name** | **Mean phenotype** | **Delta%** | Lineage | **QTL name** | **Mean phenotype** | **Delta%** |
| --- | --- | --- | --- | --- | --- | --- | --- | --- |
|  |  |  |  |  |  |  |  |  |
| **Shared** | Both | *pf7.2** | 0.525 | -38.5 | Both | *sss1.2.1*** | 32.4ł | -47.0 |
|  |  | *pf7.2*** | 0.785 | -12.8 |  |  |  |  |
|  |  |  |  |  |  |  |  |  |
| **Lineage-specific** | SH | *pf1.1* | 0.56 | -33.3 | SH | *sss4.1* | 20.1 | -63.8 |
|  | SH | *pf2.1** | 0.49 | -41.7 | SH | *sss5.1* | 9 | -83.8 |
|  |  | *pf2.1*** | 0.83 | -12.6 | SH | *sss8.1* | 10.4 | -81.3 |
|  | SH | *pf4.1* | 0.58 | -31.0 | SP | *sss1.1* | 20.97 | -70.7 |
|  | SH | *pf6.1* | 0.45 | -46.4 | SP | *sss2.1** | 26.82 | -62.5 |
|  | SH | *pf7.1* | 0.59 | -29.8 |  | *sss2.1*** | 11.08 | -81.8 |
|  | SH | *pf9.1** | 0.58 | -31.0 | SP | *sss4.1* | 21 | -70.6 |
|  |  | *pf9.1*** | 0.45 | -52.6 | SP | *sss1.2.2*** | 19.5ł | -68.0 |
|  | SH | *pf10.1* | 0.49 | -41.7 |  |  |  |  |
|  | SP | *pf1.1* | 0.57 | -41.8 |  |  |  |  |
|  | SP | *pf3.1* | 0.63 | -27.1 |  |  |  |  |
|  | SP | *pf4.1* | 0.58 | -33.4 |  |  |  |  |
|  | SP | *pf8.1* | 0.53 | -52.4 |  |  |  |  |
|  | SP | *pf9.1** | 0.66 | -31.7 |  |  |  |  |
|  |  | *pf9.1*** | 0.71 | -25.3 |  |  |  |  |
|  | SP | *pf11.1* | 0.67 | -26.9 |  |  |  |  |
|  |  |  |  |  |  |  |  |  |
| **Lineage-specific:** | SH | All late* | 0.53 | -36.4 | SH | All late* | 13.17 | -76.3 |
| **Means over all loci** | SP | All late* | 0.61 | -35.6 | SP | All late* | 22.07 | -68.0 |
|  | SH & SP | All late* | 0.57 | -36.0 | SH & SP | All late* | 17.62 | -72.1 |
|  | SH | All late** | 0.56 | -35.4 | SH | All late** | 13.17 | -76.3 |
|  | SP | All late** | 0.62 | -34.5 | SP | All late** | 18.14 | -72.8 |
|  | SH & SP | All late** | 0.59 | -34.9 | SH & SP | All late** | 15.65 | -74.5 |
