# Supplementary material for: Interspecific Tests of Allelism Reveal the Evolutionary Timing and Pattern of Accumulation of Reproductive Isolation Mutations
Source: PLoS Genet. 2014 Sep 11;10(9):e1004623. doi: 10.1371/journal.pgen.1004623 (PMC4161300; doi:10.1371/journal.pgen.1004623)
Supplement: Table S4 — Multivariate analyses of variance of seed fertility in introgression line genotypes at four reproductive isolation QTL, taking into account pollen fertility in the same genotypes. Effects are genotype, maternal family nested within genotype, and pollen fertility (PF); significant effects are in bold. Least squares means contrasts are based on Tukey's Honest Significant Difference (HSD) (i.e. corrected for multiple tests). (DOCX) [file pgen.1004623.s007.docx]

**Table S4**: Multivariate analyses of variance of seed fertility in introgression line genotypes at four reproductive isolation QTL, taking into account pollen fertility in the same genotypes. Effects are genotype, maternal family nested within genotype, and pollen fertility (PF); significant effects are in bold. Least squares means contrasts are based on Tukey's Honest Significant Difference (HSD) (i.e. corrected for multiple tests).

|  | **Whole Model** | |  | |  | |  |  | |  | **Effect tests** |  |  |  |  | **Contrasts** |  |  |  |
| --- | --- | --- | --- | --- | --- | --- | --- | --- | --- | --- | --- | --- | --- | --- | --- | --- | --- | --- | --- |
| **QTL** | **Source** | | **DF** | | **SS** | | **MS** | **F** | | **P** | **Source** | **DF** | **SS** | **F** | **P** | **Genotype** | **Least Square Mean** | **Std Error** | **HSD** |
| *sss1.2* | Model | | 8 | | 33519.879 | | 4189.98 | 30.4714 | | <0.0001 | Genotype | 3 | 29734.493 | 72.0807 | **<0.0001** | SL | 60.559869 | 2.3327 | A |
|  | Error | | 79 | | 10862.94 | | 137.51 |  | |  | Geno(MatFam) | 4 | 1374.104 | 2.4983 | **0.0492** | IL_PP | 10.974347 | 2.41202 | C |
|  |  | |  | |  | |  |  | |  | PF | 1 | 137.873 | 1.0027 | 0.3197 | IL_HH | 38.08421 | 2.8444 | B |
|  |  | |  | |  | |  |  | |  |  |  |  |  |  | IL_HP | 25.895164 | 6.0339 | BC |
|  |  | |  | |  | |  |  | |  |  |  |  |  |  | IL_PH | - | - | - |
| *sss2.1* | Model | | 7 | | 43679.751 | | 6239.96 | 21.7524 | | <0.0001 | Genotype | 3 | 35077.979 | 40.7605 | **<0.0001** | SL | 54.097962 | 3.5168133 | B |
|  | Error | | 79 | | 22662.149 | | 286.86 |  | |  | Geno(MatFam) | 3 | 2314.168 | 2.6891 | 0.052 | IL_PP | 11.477333 | 4.6979224 | C |
|  |  | |  | |  | |  |  | |  | PF | 1 | 11169.455 | 38.9366 | **<0.0001** | IL_HH | 68.869895 | 3.32147 | A |
|  |  | |  | |  | |  |  | |  |  |  |  |  |  | IL_HP | 72.999867 | 3.9695184 | A |
|  |  | |  | |  | |  |  | |  |  |  |  |  |  | IL_PH | - | - | - |
| *pf7.2* | Model | | 9 | | 3153.966 | | 350.441 | 8.15 | | <0.0001 | Genotype | 4 | 2971.2213 | 17.275 | **<0.0001** | SL | 20.988438 | 1.1944953 | A |
|  | Error | | 188 | | 8083.802 | | 42.999 |  | |  | Geno(MatFam) | 4 | 135.9957 | 0.7907 | 0.5325 | IL_PP | 9.616188 | 0.9677557 | B |
|  |  | |  | |  | |  |  | |  | PF | 1 | 171.3844 | 3.9858 | **0.0473** | IL_HH | 9.552796 | 0.9789412 | B |
|  |  | |  | |  | |  |  | |  |  |  |  |  |  | IL_HP | 10.572532 | 1.1614995 | B |
|  |  | |  | |  | |  |  | |  |  |  |  |  |  | IL_PH | 10.870488 | 1.1287371 | B |
| *pf9.1* | Model | | 8 | | 70012.21 | | 8751.53 | 8.3327 | | <0.0001 | Genotype | 4 | 62676.896 | 14.9193 | **<0.0001** | SL | 54.17357 | 7.5051 | BC |
|  | Error | | 85 | | 89272.62 | | 1050.27 |  | |  | Geno(MatFam) | 3 | 1542.626 | 0.4896 | 0.6904 | IL_PP | 41.50532 | 8.3718 | C |
|  |  | |  | |  | |  |  | |  | PF | 1 | 3037.05 | 2.8917 | 0.0927 | IL_HH | 113.17495 | 7.7333 | A |
|  | |  | |  | |  |  | |  |  |  |  |  |  |  | IL_HP | 114.50117 | 8.5703 | A |
|  | |  | |  | |  |  | |  |  |  |  |  |  |  | IL_PH | 75.44582 | 8.4028 | B |
