# Supplementary material for: Interspecific Tests of Allelism Reveal the Evolutionary Timing and Pattern of Accumulation of Reproductive Isolation Mutations
Source: PLoS Genet. 2014 Sep 11;10(9):e1004623. doi: 10.1371/journal.pgen.1004623 (PMC4161300; doi:10.1371/journal.pgen.1004623)
Supplement: Table S5 — Effect sizes of DMIs associated with earlier evolving versus later evolving sterility-causing mutations. delta% describes the proportional reduction in fertility in comparison to the fully fertile recurrent parent (SL), as in [7], [27]. Bootstrap analysis was performed on delta% values because these account for differences in average fertility of the SL genotype between different experiments (Text S1). Analysis results are given for effect size comparisons on two possible cuts of the dataset for each of our fertility traits: the first preferentially uses effect size estimates from the original analyses (where possible) and the second preferentially uses estimates from the analyses here (where possible) (Text S2). (DOCX) [file pgen.1004623.s008.docx]

**Table S5**: Effect sizes of DMIs associated with earlier evolving versus later evolving sterility-causing mutations. delta% describes the proportional reduction in fertility in comparison to the fully fertile recurrent parent (SL), as in [7], [27]. Bootstrap analysis was performed on delta% values because these account for differences in average fertility of the SL genotype in the different experiments (Text S1). Analyses results are given for effect size comparisons on two possible cuts of the dataset for each of our fertility traits: the first preferentially uses effect size estimates from the original analyses (where possible) and the second preferentially uses estimates from the analyses here (where possible) (Text S2).

|  | **Effect sizes estimated from:** | **Previous experiments (where possible)** | | **This experiment (where possible)** | |
| --- | --- | --- | --- | --- | --- |
|  |  | **PF** | **SSS** | **PF** | **SSS** |
| **Mean Phenotype** | Early | 0.525 | 32.40 | 0.785 | 32.40 |
|  | Late: SH-specific | 0.534 | 13.17 | 0.564 | 13.17 |
|  | Late: SP-specific | 0.607 | 22.07 | 0.615 | 18.14 |
|  | Late: all loci | 0.570 | 17.62 | 0.590 | 15.65 |
| **Mean Delta%** | Early | -38.50 | **-47.00** | **-12.80** | **-47.00** |
|  | Late: SH-specific | -36.41 | -76.30 | -35.35 | -76.30 |
|  | Late: SP-specific | -35.55 | -67.95 | -34.48 | -72.77 |
|  | Late: all loci | -35.98 | -72.13 | -34.91 | -74.54 |
| **Bootstrap Simulation** | Mean Delta% | -36.02 | -71.53 | -34.95 | -74.28 |
| **of all Late loci** | SE Delta% | 2.205 | 3.091 | 3.209 | 2.976 |
| **(N=500)** | 95% CI | 4.323 | 6.059 | 6.289 | 5.832 |
|  | lower 2.5% | -40.26 | **-77.63** | **-40.62** | **-79.11** |
|  | upper 97.5% | -32.62 | **-66.76** | **-29.63** | **-68.76** |
