# Supplementary material for: Interspecific Tests of Allelism Reveal the Evolutionary Timing and Pattern of Accumulation of Reproductive Isolation Mutations
Source: PLoS Genet. 2014 Sep 11;10(9):e1004623. doi: 10.1371/journal.pgen.1004623 (PMC4161300; doi:10.1371/journal.pgen.1004623)
Supplement: Table S6 — Tests of the distribution of observed QTL on early versus late branches, contingent upon observed branch lengths, using χ2 goodness-of-fit tests. Two different models of incompatibility accumulation are evaluated: ‘linear’ and ‘exponential’ models. Two cases that evaluate uncertainty in branch length are also included (Text S2). ‘Extrapolated loci’ indicates the case in which we assume that 1/3 of our lineage-specific QTL are underpinned by two mutations. Significant comparisons are in bold. (DOCX) [file pgen.1004623.s009.docx]

**Table S6**: Tests of the distribution of observed QTL on early versus late branches, contingent upon observed branch lengths, using Chi2 goodness-of-fit tests. Two different models of incompatibility accumulation are evaluated: ‘linear’ and ‘exponential’ models. Two cases that evaluate uncertainty in branch length are also included (Text S2). 'Extrapolated loci' indicates the case in which we assume that 1/3 of our lineage-specific QTL are underpinned by two mutations. Significant comparisons are in bold.

|  | **DMIs** |  | **Pollen Sterility** |  |  | **Seed Sterility** |  |  |
| --- | --- | --- | --- | --- | --- | --- | --- | --- |
| **Empirical estimates of branch lengths** |  |  | **Observed** | **Linear model: expected** | **Exponential model: expected** | **Observed** | **Linear model: expected** | **Exponential model: expected** |
| (p_early:p_late) = (0.251:0.749) | Observed loci | Late Loci | 13.000 | 10.481 | 13.116 | 7.000 | 5.989 | 7.495 |
|  |  | Early Loci | 1.000 | 3.519 | 0.884 | 1.000 | 2.011 | 0.505 |
|  |  | Total | 14.000 | 14.000 | 14.000 | 8.000 | 8.000 | 8.000 |
|  |  | Chi-Sq |  | 2.408 | 0.016 |  | 0.508 | 0.517 |
|  |  | p |  | NS | NS |  | NS | NS |
| (p_early:p_late) = (0.251:0.749) | Extrapolated loci | Late Loci | 17.333 | 13.725 | 17.175 | 9.333 | 7.736 | 9.681 |
|  |  | Early Loci | 1.000 | 4.608 | 1.158 | 1.000 | 2.597 | 0.653 |
|  |  | Total | 18.333 | 18.333 | 18.333 | 10.333 | 10.333 | 10.333 |
|  |  | Chi-Sq |  | 3.773 | 0.023 |  | 1.312 | 0.197 |
|  |  | p |  | **0.050** | NS |  | NS | NS |
| (p_early:p_late) = (0.251:0.749) | Minimum ratio for a significant Chi2 | Late Loci | 12.000 | 8.984 | 11.242 |  |  |  |
|  |  | Early Loci | 0.000 | 3.016 | 0.758 |  |  |  |
|  |  | Total | 12.000 | 12.000 | 12.000 |  |  |  |
|  |  | Chi-Sq |  | 4.029 | 0.809 |  |  |  |
|  |  | p |  | **<0.05** | NS |  |  |  |
| **Estimates considering uncertainty in branch lengths** | |  |  |  |  |  |  |  |
| Case A: Internal branch length underestimated | Observed loci | Late Loci | 13.000 | 9.919 | 12.810 | 7.000 | 5.668 | 7.320 |
| (p_early:p_late) = (0.291:0.709) | | Early Loci | 1.000 | 4.081 | 1.190 | 1.000 | 2.332 | 0.680 |
|  |  | Total | 14.000 | 14.000 | 14.000 | 8.000 | 8.000 | 8.000 |
|  |  | Chi-Sq |  | 3.283 | 0.033 |  | 1.074 | 0.165 |
|  |  | p |  | **0.10>p>0.05** | NS |  | NS | NS |
| Case A | Extrapolated loci | Late Loci | 17.333 | 12.989 | 16.776 | 9.333 | 7.321 | 9.455 |
|  |  | Early Loci | 1.000 | 5.344 | 1.558 | 1.000 | 3.012 | 0.878 |
|  |  | Total | 18.333 | 18.333 | 18.333 | 10.333 | 10.333 | 10.333 |
|  |  | Chi-Sq |  | 4.984 | 0.218 |  | 1.897 | 0.019 |
|  |  | p |  | **<0.05** | NS |  | NS | NS |
|  |  |  |  |  |  |  |  |  |
| Case B: Internal branch length overestimated | Observed loci | Late Loci | 13.000 | 11.111 | 13.404 | 7.000 | 6.349 | 7.659 |
| (p_early:p_late) = (0.206:0.794) | | Early Loci | 1.000 | 2.889 | 0.596 | 1.000 | 1.651 | 0.341 |
|  |  | Total | 14.000 | 14.000 | 14.000 | 8.000 | 8.000 | 8.000 |
|  |  | Chi-Sq |  | 1.556 | 0.286 |  | 0.323 | 1.333 |
|  |  | p |  | NS | NS |  | NS | NS |
| Case B | Extrapolated loci | Late Loci | 17.333 | 14.550 | 17.553 | 9.333 | 8.201 | 9.893 |
|  |  | Early Loci | 1.000 | 3.783 | 0.781 | 1.000 | 2.132 | 0.440 |
|  |  | Total | 18.333 | 18.333 | 18.333 | 10.333 | 10.333 | 10.333 |
|  |  | Chi-Sq |  | 2.580 | 0.064 |  | 0.758 | 0.744 |
|  |  | p |  | NS | NS |  | NS | NS |
