# Supplementary material for: Interspecific Tests of Allelism Reveal the Evolutionary Timing and Pattern of Accumulation of Reproductive Isolation Mutations
Source: PLoS Genet. 2014 Sep 11;10(9):e1004623. doi: 10.1371/journal.pgen.1004623 (PMC4161300; doi:10.1371/journal.pgen.1004623)
Supplement: Table S7 — Tests of the distribution of observed QTL on early versus late branches, contingent upon observed branch lengths (‘RAxML’), using binomial resampling. For comparison, expectations and results from the same tests—but assuming equal branch lengths on branches b, c, and d in the three species phylogeny (‘Equal’)–are also shown. Significant comparisons are in bold. For each, expected pearly = (1-plate). All bootstrap simulations performed with N = 1000. ‘Extrapolated # loci’ indicates the case in which we assume that 1/3 of our lineage-specific QTL are underpinned by two mutations. * rounded to nearest whole number. (DOCX) [file pgen.1004623.s010.docx]

**Table S7**: Tests of the distribution of observed QTL on early versus late branches, contingent upon observed branch lengths (‘RAxML’), using binomial resampling. For comparison, expectations and results from the same tests—but assuming equal branch lengths on branches b, c, and d in the three species phylogeny (‘Equal’)--are also shown. Significant comparisons are in bold. For each, expected p_early_ = (1-p_late_). All bootstrap simulations performed with N=1000. 'Extrapolated # loci' indicates the case in which we assume that 1/3 of our lineage-specific QTL are underpinned by two mutations.

* rounded to nearest whole number

|  |  |  |  |  | **p_late** | | **Prop. simulations that show a more extreme distribution than the observed distribution** | |
| --- | --- | --- | --- | --- | --- | --- | --- | --- |
|  | **Trait** | **Late loci** | **Total loci** | **Branch length model** | **Linear** | **Exponential** | **Linear** | **Exponential** |
| Observed # loci | PF | 13 | 14 | RAxML | 0.7486 | 0.937 | 0.086 | 0.782 |
|  |  | 13 | 14 | Equal | 0.6667 | 0.88888 | **0.028** | 0.515 |
|  | SSS | 7 | 8 | RAxML | 0.7486 | 0.937 | 0.322 | 0.913 |
|  |  | 7 | 8 | Equal | 0.6667 | 0.88888 | 0.2 | 0.787 |
| Extrapolated # loci* | PF | 17 | 18 | RAxML | 0.7486 | 0.937 | **0.039** | 0.668 |
|  |  | 17 | 18 | Equal | 0.6667 | 0.88888 | **0.005** | 0.385 |
|  | SSS | 9 | 10 | RAxML | 0.7486 | 0.937 | 0.25 | 0.877 |
|  |  | 9 | 10 | Equal | 0.6667 | 0.88888 | 0.094 | 0.687 |
