# Supplementary material for: Interspecific Tests of Allelism Reveal the Evolutionary Timing and Pattern of Accumulation of Reproductive Isolation Mutations
Source: PLoS Genet. 2014 Sep 11;10(9):e1004623. doi: 10.1371/journal.pgen.1004623 (PMC4161300; doi:10.1371/journal.pgen.1004623)
Supplement: Table S8 — Comparative QTL results for the original mapping studies (‘previous threshold’) and under two more permissive statistical thresholds for detecting QTL in each mapping population, for A) pollen fertility and B) seed fertility QTL. MP = moderately permissive threshold; HP = highly permissive threshold (Text S1). QTL are listed in linear order along chromosomes (1 through 12); detected loci that are physically co-localized with a QTL detected in the other species cross are identified in the final column for each population. New QTL detected only under one or both more permissive statistical thresholds are shown in bold; note that in most cases, new ILs found to be statistically significant under more permissive thresholds coincided with a chromosomal location where a significant QTL had already been detected in an overlapping IL from the same population under a more stringent statistical cut-off. * sss loci that were not statistically independent of associated pollen sterility effects. **for consistency between the two mapping studies, this locus is re-labeled pf7.2 in the current study. ***sss7.1 is co-localized with, and statistically dependent upon, pf7.2. ł one line supports two previously identified QTL. łł unknown which of these are independent of pollen sterility. (DOCX) [file pgen.1004623.s011.docx]

**Table S8**: Comparative QTL results for the original mapping studies (‘previous threshold’) and under two more permissive statistical thresholds for detecting QTL in each mapping population, for A) pollen fertility and B) seed fertility QTL. MP = moderately permissive threshold; HP = highly permissive threshold (Text S1). QTL are listed in linear order along chromosomes (1 through 12); detected loci that are physically co-localized with a QTL detected in the other species cross are identified in the final column for each population. New QTL detected only under one or both more permissive statistical thresholds are shown in bold; note that in most cases, new ILs found to be statistically significant under more permissive thresholds coincided with a chromosomal location where a significant QTL had already been detected in an overlapping IL under a more stringent statistical cut-off.

* sss loci that were not statistically independent of associated pollen sterility effects

**for consistency between the two mapping studies, this locus is re-labeled *pf7.2* in the current study.

****sss7.1* is colocalized with, and statistically dependent upon, *pf7.2*

ł one line supports two previously identified QTL

łł unknown which of these are independent of pollen sterility

|  | **Population 1: SL x SH** | | | **# ILs supporting** | | |  | | **Population 2: SL x SP** | | | **# ILs supporting** | | |  |
| --- | --- | --- | --- | --- | --- | --- | --- | --- | --- | --- | --- | --- | --- | --- | --- |
| **Trait** | **Csome** | | **QTL name** | **Previous Threshold** | **MP Threshold** | **HP Threshold** | **Colocalized (QTL in other population)** | | **Csome** | | **QTL name** | **Previous Threshold** | **MP Threshold** | **HP Threshold** | **Colocalized (QTL in other population)** |
| PF | 1 | | pf1.1 | 1 | 1 | 1 | N |  | | 1 | pf1.1 | 2 | 2 | 2 | N |
| PF | 2 | | pf2.1 | 1 | 1 | 1 | N |  | | 3 | pf3.1 | 1 | 1 | 1 | N |
| PF | 4 | | pf4.1 | 1 | 1 | 1 | N |  | | 4 | pf4.1 | 1 | 1 | 2 | N |
| PF | 6 | | pf6.1 | 1 | 1 | 1 | N |  | | 7 | pf7.1** | 1 | 1 | 1 | Y (pf7.2) |
| PF | 7 | | pf7.1 | 1 | 2 | 2 | N |  | | 8 | pf8.1 | 1 | 2 | 2 | N |
| PF | 7 | | pf7.2 | 3 | 3 | 4 | Y (pf7.2) |  | | 9 | pf9.1 | 1 | 1 | 1 | N |
| PF | 9 | | pf9.1 | 1 | 1 | 1 | N |  | | 11 | pf11.1 | 1 | 1 | 1 | N |
| PF | 10 | | pf10.1 | 3 | 3 | 4 | N |  | |  |  |  |  |  |  |
| PF | 3 | | **NEW** | 0 | 0 | 1 | **N** |  | | 2 | **NEW** | 0 | 1 | 1 | **N** |
| PF | 9 | | **NEW** | 0 | 0 | 2 | **Y (pf9.1)** |  | | 9 | **NEW** | 0 | 0 | 1 | **Y (pf9.1)** |
|  |  | |  |  |  |  |  |  | | 10 | **NEW** | 0 | 1 | 1 | **N** |
|  |  | | Total ILs signif. | 12 | 13 | 18 |  |  | |  | Total ILs signif. | 8 | 11 | 13 |  |
|  |  | | Total QTL | 8 | 8 | 10 |  |  | |  | Total QTL | 7 | 9 | 10 |  |
|  |  | | Unique QTL | 7 | 7 | 8 |  |  | |  | Unique QTL | 6 | 8 | 8 |  |
|  |  | | Shared QTL | 1 | 1 | 2 |  |  | |  | Shared QTL | 1 | 1 | 2 |  |
| SSS | 2 | | sss1.2 | 3 | 4 | 6 | Y (sss1.2) |  | | 1 | sss1.1 | 1 | 1 | 1 | N |
| SSS | 4 | | sss4.1 | 1 | 1 | 1 | N |  | | 1 | sss1.2 | 1 | 1 | 1 | Y (sss1.2) |
| SSS | 5 | | sss5.1 | 1 | 1 | 1 | N |  | | 2 | sss2.1 | 1 | 2 | 2 | Y (sss2.1*) |
| SSS | 8 | | sss8.1 | 1 | 1 | 2 | N |  | | 4 | sss4.1 | 1 | 1 | 1 | N |
| SSS | 1 | | sss1.1* | 1 | 1 | 2 | N |  | | 7 | sss7.1* | 1 | 1 | 1 | Y (sss7.1*)*** |
| SSS | 3 | | sss2.1* | 1 | 1 | 1 | Y (sss2.1) |  | | 8 | sss8.1* | 1 | 1 | 1 | N |
| SSS | 4 | | sss4.2* | 2 | 4 | 5 | N |  | | 9 | sss9.1* | 1 | 1 | 1 | N |
| SSS | 7 | | sss7.1* | 1 | 2 | 2 | Y (sss7.1*)*** | |  | |  |  |  |  |  |
| **SSS** | **5** | | **NEW** | **0** | **0** | **3** | **N** |  | | **3** | **NEW** | **0** | **1** | **1** | **N** |
| **SSS** | **6** | | **NEW** | **0** | **0** | **1** | **N** |  | | **8** | **NEW** | **0** | **0** | **1** | **N** |
| **SSS** | **9** | | **NEW** | **0** | **0** | **1** | **Y (sss9.1)** |  | |  |  |  |  |  |  |
| **SSS** | **10** | | **NEW** | **0** | **0** | **2** | **N** |  | |  |  |  |  |  |  |
|  | |  | Total ILs signif. | 10 | 15 | 26ł |  |  | |  | Total ILs signif. | 7 | 9 | 10ł |  |
| **Only SSS QTL** | | | Total QTL | 4 | 4 | 8łł |  |  | |  | Total QTL | 4 | 5 | 6łł |  |
| **indep. of PF** | |  | Unique QTL | 2 | 2 | 5 |  |  | |  | Unique QTL | 2 | 3 | 4 |  |
| **plus NEW QTL** | | | Shared QTL | 2 | 2 | 3 |  |  | |  | Shared QTL | 2 | 2 | 2 |  |
|  | |  |  |  |  |  |  |  | |  |  |  |  |  |  |
| All QTL detected | | | Total QTL | 8 | 8 | 12 |  |  | |  | Total QTL | 7 | 8 | 9 |  |
| with signif. | |  | Unique QTL | 6 | 6 | 9 |  |  | |  | Unique QTL | 5 | 6 | 7 |  |
| SSS effects | |  | Shared QTL | 2 | 2 | 3 |  |  | |  | Shared QTL | 2 | 2 | 2 |  |
