# Supplementary material for: Interspecific Tests of Allelism Reveal the Evolutionary Timing and Pattern of Accumulation of Reproductive Isolation Mutations
Source: PLoS Genet. 2014 Sep 11;10(9):e1004623. doi: 10.1371/journal.pgen.1004623 (PMC4161300; doi:10.1371/journal.pgen.1004623)
Supplement: Table S9 — Primer pairs used for additional genotyping on chromosome 7. For marker TG61, primer sequences on SolGenomics were used. (DOCX) [file pgen.1004623.s012.docx]

**Table S9**: Primer pairs used for additional genotyping on chromosome 7. For marker TG61, primer sequences on SolGenomics were used.

| **Marker ID** | **Marker Type** | **Forward Primer** | **Reverse Primer** | **Repeat** | **Source Reference*** |
| --- | --- | --- | --- | --- | --- |
| TES1008 | EST-SSR | GACTAGCAAAGCAGAGCGGAG | AAATGGCGTTATGTCAAGGC | AAG | Shirasawa et al 2010 TAG |
| TES0584 | EST-SSR | CGAAAGAACCCTAGCCAAGA | GACCACTAGGGCTCAACGAG | AAT | Shirasawa et al 2010 TAG |
| TES0881 | EST-SSR | GATTTTGGAAAAATCGCCCTT | CATCGAAGACGGAACATCCT | AT | Shirasawa et al 2010 TAG |
| SSR286 | Genomic-SSR | AGCTATGGAGTTTCAGGACCA | ATTCAGGTAGCATGGAACGC | CAG | Sol-Genomics |
| TG61 | RFLP |  |  |  | Sol-Genomics |
| TES0181 | EST-SSR | GAGCCCCATTTACAGCCTCTT | TTTTTGTTGCACCCTTCTCA | AAT | Shirasawa et al 2010 TAG |
| TES0130 | EST-SSR | GAAGCAAATCGTCTTAGCGG | CTTCTCGCACAAGTGAACCA | AAT | Shirasawa et al 2010 TAG |
| TES1075 | EST-SSR | GTCAACTCTCATTGAACCCCC | CGGAGAAGATGAGGCAAAAA | AAT | Shirasawa et al 2010 TAG |
| TES0682 | EST-SSR | GCTCTTCCCCCTGAGCTCTCT | CAAAGGCGCCAGATTTAGTC | AG | Shirasawa et al 2010 TAG |
| TES0903 | EST-SSR | GATCGATGGGAAGGCTGA | ACTTGAGCCTCTGGGAACAA | AAT | Shirasawa et al 2010 TAG |
| TES0263 | EST-SSR | GTGAGAAGATGGGGATTGGAC | TTCAGCACTCTCCTCCACCT | ATC | Shirasawa et al 2010 TAG |
| TES1718 | EST-SSR | GAGCCTAGGGTTTCTTCACCC | TGCTTCACATGTACGTTTCCA | AAG | Shirasawa et al 2010 TAG |
| TES0509 | EST-SSR | AGAAAAGAACCTCAACACAGCA | GACACCAGCTTCAGTCAGCA | AAG | Shirasawa et al 2010 TAG |
