# Supplementary material for: Interspecific Tests of Allelism Reveal the Evolutionary Timing and Pattern of Accumulation of Reproductive Isolation Mutations
Source: PLoS Genet. 2014 Sep 11;10(9):e1004623. doi: 10.1371/journal.pgen.1004623 (PMC4161300; doi:10.1371/journal.pgen.1004623)
Supplement: Text S1 — Supporting information for materials and methods, including details of plant handling and cultivation, fertility evaluation, ancillary marker genotyping, phylogenetic analysis, and assessment of QTL detection under more permissive statistical thresholds. (DOCX) [file pgen.1004623.s013.docx]

**Text S1: Supporting Materials and Methods**

**Plant material, cultivation, and handling:**  The SH (IL_HH_) and SP (IL_PP_) introgression lines used to generate genotypes for tests of allelism were previously developed by Monforte and Tanksley (2000) and Eshed and Zamir (1994, 1995), respectively. Detailed descriptions of line generation are provided in previous work (see Bernacchi et et. al. 1997; Monforte and Tanksley 2000; Eshed and Zamir 1994, 1995; and summarized in Moyle and Graham 2005, Moyle and Nakazato 2008). Briefly, all lines are the advanced-generation backcross progeny of a single F1 plant produced by crossing either SP or SH as the male parent to SL. In each population, this F1 individual was backcrossed to the recurrent SL parental line for between 5 and 10 generations, and offspring lines genotyped with several hundred codominant molecular markers to assess the locations of SP or SH introgressions. In this study, only those lines that carry a co-localized QTL pair were used in tests of allelism (Table S1).

For all experimental individuals, seeds and plants were handled as in previous experiments (Moyle and Graham 2005, Moyle and Nakazato 2008). Briefly seeds were treated in 0.125% sodium hypochloride for 30 minutes, rinsed, place on blotting paper and incubated (12 hour day length, 24^O^C) to stimulate germination. Seeds that did not germinate within 5 days had the seed coat nicked at the hypocotyl end. Seedlings that were not able to completely emerge from the seed coat had the seed coat gently removed. After 10 days, germinated seedlings were transplanted to flats filled with Metro Mix 360 (Sun Gro) soilless potting mix, and hand watered daily. Three weeks after transplant, seedlings were transferred to individual two gallon pots containing a 50/50 mix of Metro Mix 360 and IU greenhouse potting mix, and put in a climate controlled greenhouse at Indiana University Bloomington (14 hour day). Plants were watered daily by drip irrigation, fertilized twice weekly, and pruned and staked prior to flowering.

To generate IL_HP_ and IL_PH_ lines, 3-5 individuals of parental IL_HH_ and IL_PP_ lines were germinated and grown to flowering. On each maternal (seed) parent, flowers were hand-emasculated and, after 24 hours, hand-pollinated with pollen from the appropriate donor parent. For example, IL_HP_ seeds were generated by pollinating IL_HH_ flowers with IL_PP_ pollen. Simultaneous with generating heterospecific introgression lines, plants were also self-pollinated to generate IL_HH_ and IL_PP_ seeds for the main experiment. Fruits were allowed to develop until maturity, collected, and seeds extracted by hand.

For the main experiment, for each QTL examined, at least 25 seeds were germinated for each of IL_HP_, IL_PH_, IL_PP_, IL_HH_, and SL genotypes. Each plant was assigned a unique ID and was grown to flowering.

**Fertility evaluation of PF and SSS:** Fertility was evaluated as for both previous QTL mapping experiments with these lines (Moyle and Graham 2005, Moyle and Nakazato 2008). Pollen fertility (PF) was estimated on each of three unopened flowers on each plant per Moyle and Graham (2005). Briefly, anther cones from each of three flowers were individually collected into eppendorf tubes containing lactophenol-aniline blue histochemical stain, which is routinely used to assess pollen fertility (Kearns and Inouye 1993). In each sample, proportion fertile pollen (PF), was estimated by counting aliquot subsamples of pollen on haemocytometer microscope slides. Pollen inviability was indicated by a lack of stained cytoplasm; this is a conservative measure of pollen infertility as some grains that stain for cytoplasm may be functionally inviable for other reasons (Kearns and Inouye 1993). For each plant, PF was estimated as the mean of the three pollen samples from that plant.

Seed fertility (SSS) was estimated as seeds per hand-pollinated fruit. For each experimental individual, eight freshly opened flowers were hand pollinated with pollen from the same individual and tagged. At maturity, fruit was harvested, seeds extracted by hand from individual fruits, and seed fertility determined by counting the number of plump, filled seeds. For each plant, SSS was estimated as the average number of seeds per fruit from the first four hand-pollinated fruits to develop to maturity.

**Marker genotyping to more finely delimit introgression breakpoints for *pf7.2*:**

Genotyping with 9 markers on chromosome 7 more finely determined the chromosomal locations of introgressions corresponding to *pf7.2* in each mapping population, and confirmed the degree of overlap between these QTL in the two mapping populations (Figure 2). Briefly, published simple sequence repeat (SSR) markers known to differentiate SL and SP (Shirasawa et al. (2010), and solgenomics.org; Table S9), were tested to identify markers (primer pairs) that were also able to distinguished SH and SL alleles. Using standard CTAB extraction protocols, we extracted DNA from 3-6 IL_HH_, IL_PP_, and SL individuals, PCR-amplified each marker using the relevant primer pair, resolved amplification products on an ABI 3730X1 DNA Sequencer (Applied Biosystems), and called genotypes for each locus in each individual using the software package Genemapper v. 4.0 (Applied Biosystems). Resulting marker positions, map distances, and locations of introgression regions are shown in Figure 2A (main text).

**Phylogenetic analysis to infer branch lengths (mean and variance):** Details of tree construction are provided elsewhere (Haak et al. 2014). Briefly, trees were reconstructed using publicly available sequence data for 18 genes from all thirteen species in the clade *Solanum* Section *Lycopersicon*, as well as the outgroup *S. lycopersicoides*. Maximum likelihood (ML) analyses were conducted using RAxML Pthreads 7.0.0 (Stamatakis 2006) using one step (-f a, 10,000 bootstraping replicates) by applying ML tree search and rapid bootstrapping. We chose the best supported bipartitioned tree calculated from the generalized time reversible (GTR) substitution model with a GAMMA model of rate heterogeneity. The topology of the best tree agreed with previous Bayesian estimates (Rodriguez et al. 2009), in addition to our own Mr Bayes analysis (Haak et al. 2014). Variability in estimation of the phylogeny was incorporated into our subsequent analyses in two ways. For our assessment of whether isolation-causing mutations were linearly proportionate to branch lengths, standard deviations for relevant branch lengths on the RAxML tree were obtained from 100 bootstrap replicates, using the topology from the best RAxML partitioned tree (Figure S2). These estimates were used to assess the effect of significant branch length error on our assessment of whether isolation-causing mutations occur disproportionately on late branches (see Text S2). For our comparison of phylogenetic models of incompatibility accumulation, we estimated branch lengths using trees drawn from the posterior distribution of our MrBayes analysis (Haak et al. 2014). This posterior was represented by sampling every 100 generations from a Markov chain running for 10,000,000 generations, discarding the first 25% of trees as burn-in. Since the mathematical description of incompatibility accumulation assumes substitutions accumulate at a constant rate between different branches, we used a penalized-likelihood method, as implemented in R/*ape*, to enforce a molecular clock on the branch lengths drawn from the posterior. In the resulting 75,000 trees, the median estimate of relative internal and tip branch lengths (i.e. P_early_ and P_late_) are identical to the mean estimates from the RAxML tree. The fit of each iteration (N=75,000) to an accumulation model was determined using the maximum-likelihood method in Wang et al. (2013) and Akaiki Information Criterion (AIC) values were evaluated under the four different models of incompatibility accumulation; e.g. AIC_linear_(***v_DM_***), AIC_DM_(***v_DM_***), etc. Results are summarized as the mean AIC (and standard deviation) for each model, within each of the pollen and seed datasets. Overall, we find that our phylogenetically informed ML approach still strongly supports models of non-linear accumulation for both pollen and seed sterility; therefore our inference of ‘snowballing’ accumulation appears to be robust to variance in estimating the lengths of the relevant branches.

**Simulations to determine the power of model selection:** To determine our ability to distinguish between different models of incompatibility accumulation, we ran two sets of simulations, one based on the number of incompatibilities found in the pollen data and another based on the number found in the seed data. These simulations place substitutions on a random sample of our ultrametricized posterior distribution of trees from Mr Bayes (above) according to a Poisson distribution with an expected number of substitutions proportionate to the tree’s branch lengths. Potential interactions from these substitutions were enumerated based on one of four different models of incompatibility accumulation (**linear**, **DM**, **2+3**, **p_a_ ≠ p_d_**). Incompatibilities were then simulated by drawing from these potential interactions using a binomial distribution with the model’s respective probabilities, e.g. *p*, *p_2_* and *p_3_*, etc. These probabilities were scaled such that the expected number of incompatibilities in a hybrid between the most diverged taxa matches the empirical values from the pollen or seed data respectively. This ensures that comparisons between the empirical results and the simulations use a similar numbers of incompatibilities. For the 2+3 and p_a_ ≠ p_d_ simulations, a random number of incompatibilities are assigned to each process (e.g. quadratic versus cubic), but are constrained such that the expected sum is equal to the empirical value.

The output of a single iteration of the simulation is a vector of incompatibility observations, e.g. an iteration under the DM model might be ***v_DM_*** = {*I_AB_* = 5, *I_AC_* = 8, *I_BC_* = 10, *I_sharedC_* = 6, *I_sharedB_* = 4, *I_sharedA_* = 2}. We ran 10,000 such iterations under each of the four different accumulation models for both the seed and the pollen simulations, taking into account tree variability by sampling from the ultrametrized posterior distribution of trees (i.e. each simulation randomly sampled a tree from this distribution and simulated a DMI accumulation model on that tree). Since our empirical data contains only three of the six incompatibility observations, we removed the missing elements from all of our simulated vectors. In the example above, the incompatibility vector would become ***v_DM_*** = {*I_AC_* = 8, *I_BC_* = 10, *I_sharedC_* = 4}.

The fit of each iteration to an accumulation model was determined using the maximum-likelihood method in Wang et al. (2013) and parameter estimates along with AIC values were evaluated under the four different models; e.g. AIC_linear_(***v_DM_***), AIC_DM_(***v_DM_***), etc. We classified the method as successful whenever the minimum AIC matched the underlying simulation; in the example above, an iteration where AIC_DM_(***v_DM_***) < AIC_linear_(***v_DM_***), AIC_2+3_(***v_DM_***), AIC_pa≠pd_(***v_DM_***) would be classified as a success. From this binary classification scheme, we calculated the positive predictive value (precision) and true positive rate (recall) using 40,000 iterations for the pollen and seed simulation sets respectively. Results of these analyses (see main text) indicate that our inferences of the specific best-fit nonlinear models for pollen and seed sterility are likely to be robust. In particular, the 2+3 model remains the most favored model for the pollen data, even though distinguishing more complex models from the basic DM non-linear model is affected by uncertainty associated with the phylogeny.

**Methodological and inferential assumptions of cross-specific tests of allelism:** Implementing our cross-species tests of allelism relies on several assumptions about the expression of fertility phenotypes, notably that sterility effects are recessive and that combining alleles from different species will not have additional, complex genetic effects due to tri-genomic interactions. These assumptions appear to be reasonable for sterility effects at the three co-localized QTL targeted in this study. First, prior experiments demonstrated that quantitative sterility at these QTL is partially or completely recessive (Moyle and Graham 2005, Moyle and Nakazato 2008) (Table 1). Although two of three of our target loci here are not fully recessive, complementation tests can be performed in contexts where one or both mutations are only partially recessive, provided that the heterozygous phenotypes can be differentiated from the homozygous recessive (mutant) phenotype (Brenner 1974). This is the case for all three loci examined here, for which the fertility of homozygous introgression lines (IL_HH_ and IL_PP_) is significantly lower than the fertility of heterozygous introgression lines (i.e., IL_HL_ and IL_PL_, where L is the allele from the recurrent parent SL). In terms of our complementation tests, partial recessivity at *pf7.2* and *sss1.2* does not affect our substantive inferences. In particular, complete lack of complementation (*pf7.2* and *sss1.2.1*) cannot be otherwise explained as a consequence of partially recessive alleles. Incomplete recessivity at *sss1.2.2* is also consistent with our inference that the SP *sss1.2.2* allele is significantly complemented by an allele in SH (i.e., that IL_HP_ at this locus shows significant rescue (increase) in fertility in comparison to the IL_PP_ genotype). Moreover, although the IL_HP_ line is statistically indistinguishable from IL_HH_ (suggesting complete complementation), the estimated mean phenotype in the *sss1.2* transheterozygous (IL_HP_) line is ~10 seeds lower than the IL_HH_ genotype; this is qualitatively consistent with a difference between partial and complete complementation of the SP *sss1.2.2* allele by SH, although it would be inappropriate to place too much emphasis on parsing this non-significant difference.

Second, because these QTL were initially identified with introgression lines on the same species background (SL), complex sterility effects could only arise from interactions involving mutations in both SH and SP (at the specific heterospecific introgression region) that have dominant effects on the SL background. For example, there are rare scenarios that could explain the non-complementation phenotypes we see (for pf7.2 and sss1.2.2), such as the phenomenon of second-site noncomplementation (SSNC) whereby the double-heterozygotes at two different loci recapitulate a phenotype observed in recessive homozygotes at each locus (Hawley and Gilliland 2006). For example, SSNC might be due to mutations in independent loci that both reduce expression levels of some critical gene; in combination these could generate a heterozygous genotype that produces insufficient levels of this gene product, resulting in ‘combined haplo-insufficiency’. Such SSNC mutants will fail to complement each other, even though they are not allelic. (Other even more rare scenarios can arise; see Hawley and Gilliland 2006, and references therein.) However, most instances of SSNC arise as the minority cases within large screens for complementation groups, where there is no prior information about whether target loci are physically colocalized in the genome. They also remain very rare in comparison to standard cases of complementation. Therefore, we have little reason to expect these interactions explain our results, although until we identify the specific mutations responsible for sterility we cannot definitely exclude their contribution to our observed cross-species hybrids. Note that, in terms of interpreting our cross-specific tests of alleleism, complex genetic effects are most likely to lead to false positives– that is, the incorrect inference that independent mutations in the same chromosomal region are homologous. Therefore, if these effects exist, they will only influence our inference concerning shared loci (*pf7.1*, *sss1.2.1*), not loci inferred to be independent (*sss1.2.2*, *sss2.1*).

In addition, we make several assumptions regarding the detection of pollen and seed QTL in our initial QTL mapping studies, that could have important consequences for the classification of shared versus non-shared loci, and therefore our inferences of evolutionary history based on these classifications. In particular, if our previous mapping studies (Moyle and Graham 2005, Moyle and Nakazato 2008) differed in their power to detect phenotypic effects, or had low power to detect these effects, we could systematically undercount both the total number and the number of shared QTL for these traits. There is little evidence that these studies differed in their overall power to detect QTL. Both mapping populations used introgression lines with similar levels of biological replication, and both spanned approximately the same amount of evolutionary divergence between parents (Moyle and Nakazato 2008, 2010); accordingly, each detected approximately the same number of QTL for each sterility trait (Table S1), with a similar distribution of QTL effect sizes (Table S4). However, it is well recognized that QTL mapping experiments can be underpowered to detect loci with small effect sizes, and that the statistical discrimination of QTL in any single study can be subject to environmental and stochastic effects (Lynch and Walsh 1998). Given these factors, it is likely that both previous mapping studies failed to detect some QTL for both pollen and seed sterility, especially those loci with comparatively smaller effect sizes.

With respect to the current study, the most important consequence of failing to statistically detect isolation QTL is the risk of undercounting co-localized QTL: in order to be classified as co-localized in >1 species cross, a ‘shared’ QTL must be detected in both mapping populations. Therefore reduced power could systematically bias detection against colocalized QTL in favor of QTL that appear in only one species pair.

To evaluate the risk that we have failed to identify co-localized (and therefore potentially homologous) isolation QTL, we re-analyzed data from both original mapping studies, using a more permissive statistical threshold to discriminate the presence/absence of significant QTL. In the original studies, QTL for each trait were identified under an experiment-wise alpha of 0.05 (based on Dunnett’s tests; see Moyle and Nakazato 2008). We repeated analyses using two additional, more permissive, criteria: first, an experiment-wise alpha of 0.1 (a moderately more permissive threshold of detection); and second, an uncorrected alpha whereby each IL was compared to the recurrent parent genotype (SL) using uncorrected Student’s t tests (a highly permissive criteria for determining significance). Using PF and SSS QTL identified in separate analyses with these two more permissive criteria, we evaluated evidence for chromosomal co-localization of QTL for each trait in the two experiments by comparing the map positions of known marker locations between mapping populations, as done previously to evaluate colocalization (Moyle and Nakazato 2008). These analyses returned two alternative comparative QTL maps for each trait: one moderately permissive (MP) and one highly permissive (HP) (Table S8).

We found that in each case, we recovered the 13 (4) unique QTL for pollen (seed) sterility, and the 1 (2) colocalized QTL for pollen (seed) sterility already identified using the previous threshold (Table S8). In addition to these, the MP comparative map found 2 (1) additional unique QTL, and 0 (0) additional colocalized QTL for pollen (seed) sterility. The HP comparative map found 1 (4) additional unique QTL, and 1 (1) additional colocalized QTL for pollen (seed) sterility (Table S8). These results confirm that a more permissive threshold for calling significant QTL will uncover more QTL, however they do not support an inference that this would uncover disproportionately more co-localized QTL. In particular, even at very permissive statistical thresholds (HP), we only detected a total of one potential additional colocalized QTL for each of PF and SSS; in comparison, we detected between 3 and 5 additional unique QTL for each of PF and SSS.

These comparisons suggest that our inferences concerning the timing and patterns of accumulation of isolation loci, based on patterns of unique versus shared QTL between species pairs, have not been substantively affected by potential undercounting of shared/co-localized loci in our two mapping populations.

**Text S1: Citations**

Bernacchi D, Tanksley SD (1997) An interspecific backcross of *Lycopersicon esculentum* x *L. hirsutum*: Linkage analysis and a QTL study of sexual compatibility factors and floral traits. Genetics 147: 861-877.

Brenner S (1974) The genetics of *Caenorhabditis elegans*. Genetics 77: 71-94.

Eshed Y, Zamir D (1994) A genomic library of *Lycopersicon pennellii* in *Lycopersicon esculentum* - A tool for fine mapping of genes. Euphytica 79: 175-179.

Eshed Y, Zamir D (1995) An introgression line population of *Lycopersicon pennellii* in the cultivated tomato enables the idenitification and fine mapping of yield-associated QTL. Genetics 141: 1147-1162.

Haak DC, Ballenger BA, Moyle LC (2014) No evidence for phylogenetic constraint on natural defense evolution among wild tomatoes. Ecology 95:1633-1641.

Hawley RS, Gilliland WD (2006) Sometimes the result is not the answer: the truths and the lies that come from using the complementation test. Genetics 174: 5-15.

Kearns CA, Inouye DW (1993) Techniques for pollination biologists: University Press of Colorado. 586 p.

Monforte AJ, Tanksley SD (2000) Development of a set of near isogenic and backcross recombinant inbred lines containing most of the *Lycopersicon hirsutum* genome in a *L. esculentum* genetic background: A tool for gene mapping and gene discovery. Genome 43: 803-813.

Moyle LC, Graham EB (2005) Genetics of hybrid incompatibility between *Lycopersicon esculentum* and *L. hirsutum*. Genetics 169: 355-373.

Moyle LC, Nakazato T (2008) Comparative genetics of hybrid incompatibility: Sterility in two Solanum species crosses. Genetics 179: 1437-1453.

Rodriguez F, Wu F, Ane C, Tanksley S, Spooner DM (2009) Do potatoes and tomatoes have a single evolutionary history, and what proportion of the genome supports this history? BMC Evolutionary Biology 9.

Shirasawa K, Asamizu E, Fukuoka H, Ohyama A, Sato S, et al. (2010) An interspecific linkage map of SSR and intronic polymorphism markers in tomato. Theoretical and Applied Genetics 121: 731-739.

Stamatakis A (2006) RAxML-VI-HPC: Maximum likelihood-based phylogenetic analyses with thousands of taxa and mixed models. Bioinformatics 22: 2688-2690.

Wang RJ, Ane C, Payseur BA (2013) The evolution of hybrid incompatibilities along a phylogeny. Evolution 67: 2905-2922.
