# Supplementary material for: Interspecific Tests of Allelism Reveal the Evolutionary Timing and Pattern of Accumulation of Reproductive Isolation Mutations
Source: PLoS Genet. 2014 Sep 11;10(9):e1004623. doi: 10.1371/journal.pgen.1004623 (PMC4161300; doi:10.1371/journal.pgen.1004623)
Supplement: Text S2 — Supporting information for results, including pollen/seed multivariate analyses, QTL effect size comparisons, inferences about the phylogenetic placement of loci, evidence for the unequal distribution of sterility-causing mutations over time, and additional sterility phenotypes not associated with tests of allelism. (DOCX) [file pgen.1004623.s014.docx]

**Text S2: Supporting Results**

***1) Relationship between seed and pollen fertility effects at pf7.2 and other QTL***

In addition to detecting reduced pollen fertility at *pf7.2*, we also found that this locus is associated with reduced seed fertility (average ~49% reduction in the number of seeds produced per self-fertilized fruit; Table S2), and that patterns of seed fertility are similarly consistent with a shared allele between SH and SP (Table S2). In each of the original mapping experiments, seed sterility at this QTL (designated *sss7.1* in each experiment) was not statistically separable from pollen sterility, leading us to infer that seed sterility phenotypes were likely due to the indirect consequence of pollen sterility (because self pollen is used to generate self-fertilized seeds) rather than to an independent, physically adjacent seed-specific locus.

In these previous studies, to statistically assess independence between pollen and seed sterility within our mapping experiments, we performed a stepwise analysis whereby the pollen sterility effect on seed fertility was first evaluated (via standard linear regression) and then, using the residual values, the effect of genotype on seed sterility was assessed (see Moyle and Nakazato 2008).. In the present experiment, we used a multivariate analysis on seed fertility data that included pollen fertility as a covariate. For *pf7.2*, we detected a significant effect of PF (arcsine-squareroot transformed) (F=3.986, P=0.047) as well as a significant effect of genotype (F=17.28, P = <0.0001) (Table S3). In addition, Tukey HSD contrasts indicated that seed fertility of both homozygous ILs and of transheterospecific ILs remained significantly lower than the SL recurrent parent (data not shown). These results differ from our original analyses, because they suggest seed sterility effects that are independent of pollen sterility at the *pf7.2* introgression region. This difference might be due to several factors. In particular, because we have much higher replication per genotype (N=10-25) in the current study, our analysis here has greater power to detect seed sterility effects if they exist. Further fine mapping will be needed to definitively resolve the relationship between pollen and seed sterility at this locus.

For completeness, we also performed these analyses to examine the relationship between detected seed and pollen fertility effects (if any) at each of the other QTL in the study (Table S3). None of the substantive results reported for seed fertility phenotypes at these other loci (see main text) changed under this alternative analysis.

Moreover, as expected, *sss1.2* has no pollen fertility effect in our experiment (Table S2, Table S3); this locus was not associated with reduced pollen fertility in either previous QTL mapping study (Moyle and Graham 2005, Moyle and Nakazato 2008) (Table 1, Table S1). This differs from our observations at *sss2.1* (as discussed in the main text), for which we detected significant pollen sterility associated with the SH allele (i.e. IL_HH_ genotype) but not with the SP allele (IL_PP_ genotype) (Table S2).

***2) QTL effect sizes for early versus late evolving loci***

We assessed potential differences in effect size between earlier versus later evolving loci using a bootstrap re-sampling approach (reported in main text). This requires combining data on effect sizes that were estimated in different experiments, however effect sizes for shared/homologous loci (i.e. *sss1.2.2*, and *pf7.2*) were only estimated in the current experiment, and effect sizes for non co-localized (unique) loci were only estimated in the previous mapping experiments; in addition, for the remaining loci (i.e. those loci evaluated in this study but inferred to be non-homologous using tests of allelism) their estimated effect sizes can vary depending upon whether these values are taken from the original mapping analyses or from the current experiment (Table S4). Therefore, for completeness we performed our effect size comparisons on two possible cuts of the dataset for each of our fertility traits: the first preferentially uses effect size estimates from the original analyses (where possible) and the second preferentially uses estimates from the analyses here (where possible).

As reported in the main text, we found no evidence that late loci were on average smaller than early loci. Indeed, for both versions of the seed dataset, we found that the observed effect size associated with our earlier evolving mutation was smaller than the simulated distribution (Table S5). For the pollen dataset that preferentially used effect size estimates from the current experiment, we also found that the observed effect size associated with the earlier evolving mutation was smaller than the lower 95% CI of the simulated distribution (Table S5); the pollen dataset using effect sizes from the previous mapping experiments indicated the early mutation effect size was not outside this 95% CI. This difference is explained by the fact that we generally detected smaller pollen sterility effect sizes in the current experiment, compared to the previous studies (Moyle and Graham 2005, Moyle and Nakazato 2008); this is particularly evident for the effect size associated with the early mutation, which reduced fertility by ~39% in the previous experiment, but only by ~13% in this experiment (Table S5).

***3) Inferences about the evolutionary placement of loci inferred to be homologous: derived-derived versus derived-ancestral interactions***

Figure 5 (main text) shows our provisional assignments of all pollen (A) and seed (B) sterility loci identified between SL and each of SP and SH (listed in Suppl. Table 1), when we assign homologous loci to the evolutionary branch shared by both SP and SH after their split from their last common ancestor (LCA) with SL (i.e., branch ‘b’, Figure S2). This assignment assumes that each of the shared DMIs we detect in our analysis (*pf7.2*, *sss1.2.1*) involved a mutation that was derived in the SH/SP lineages, after divergence from their last common ancestor with SL (i.e. each of these interactions involves a derived allele in the SP/SH lineage). Note that in our crossing experiments we are only examining SH and/or SP alleles involved in this interaction; in each case, we do not know the identity or location of the interacting SL allele, although it must be present in the SL genetic background of introgression lines that show sterility phenotypes.

Alternatively, at either *sss1.2.1* or *pf7.2*, the SP/SH allele could represent ancestral allelic variation at a locus that has experienced a sterility-causing mutation along the branch leading to SL (e.g., branch ‘a’ in Figure 2, 3, 4). For this to be the case, this ancestral allele must be interacting with at least one other substitution that preceded it on the SL-specific branch; that is, sterility in this (these) case(s) is due to an ‘ancestral-derived’ interaction arising from mutations that occurred solely in the lineage leading to SL (the ‘a’ branch). Sterility expression in IL_PP_ or IL_HH_ genotypes is therefore due to the disruption of these co-selected interacting SL-specific mutations. However several lines of evidence argue against this interpretation of the SH/SP shared loci identified here, including additional information about sterility QTL from mapping experiments in other *Solanum* species pairs. In particular, between SL and two more closely related species, *S.chilense* (SC) and *S. pimpinellifolium* (SM) (Figure S2), we detect no sterility loci at the same chromosomal locations as either *sss1.2.1* or *pf7.2* (L. Moyle and E. Graham, unpubl. data; N. Sherman, S. Zhang, and L. Moyle, unpubl. data). This observation excludes the possibility that *pf7.2* and *sss1.2.1* are due to derived-ancestral interactions involving recent sequential fixations solely along the species-specific branch leading to SL (i.e. branch ‘e’, in Figure S2); otherwise other *Solanum* species should also manifest these DMIs when crossed to SL.

Alternatively, hybrids from SLxSM and SLxSC might not show sterility effects at *sss1.2.1* and *pf7.2* because all three species share the same derived alleles at these loci (that is, the relevant mutations arose and were fixed prior to their MRCA, indicated in Figure S2). For this to be the case, the detected sterility effect in SP/SH must be due to an ancestral allele (as above), and both substitutions responsible for the resulting ancestral-derived sterility interaction must have arisen and fixed after the LCA of all five species, but before the MRCA of SL, SM, and SC (Figure S2). However, the branch on which both of these substitutions must have taken place (branch ‘*f*’/shaded box; Figure S2) is extremely short. Accordingly, this scenario is much less parsimonious than one in which sterility phenotypes are due to interactions involving a derived substitution specific to the SH/SP lineage since its split from the LCA of all five species (on branch ‘*b*’; Figure S2).

Similarly, other historical scenarios can also be proposed to explain our current observations of sterility QTL involving these 5 species, but all require more complex patterns of fixation, loss, or allelic interactions, than one in which *pf7.2* and *sss1.2.1* are each due to an allele derived in SH/SP. Note that we do not currently know the chromosomal locations of isolation loci acting between SP/SH and either SM or SC, but data on these QTL would clarify the physical location and evolutionary timing of the mutation(s) with which each of our detected sterility alleles *sss1.2.1* and *pf7.1* interact.

*• Consequences of incorrect inferences about derived-derived versus ancestral-derived interactions, and therefore placement of these loci on the tree.*

Although the current evidence indicates that *sss1.2.1* and *pf7.2* arose in the shared common ancestor of SH and SP (above), several of our substantive conclusions do not strongly hinge upon this inference. For example, if these two homologous loci arose instead on the SL lineage, our inferences regarding the unequal distribution of earlier- versus later-evolving mutations involved in DMIs (section 4 below) can only become stronger. In particular, if the relevant mutation arose early in divergence along the SL branch, our current inference does not change; in comparison, if the relevant mutation(s) arose later in divergence along that lineage, these inferences become stronger, as all our detected loci are then inferred to arise on later rather than earlier branches.

Similarly, analyses involving comparisons of early versus late loci will continue to be valid if the relevant locus evolved early along the SL branch. In contrast, if each of our inferred early loci actually evolved later along the SL branch, these comparisons clearly should be used to compare the effect sizes of early versus late loci.

***4) Evidence for unequal distribution of sterility-causing mutations over time.***

Our results suggest that mutations contributing to DMIs are not equally distributed on early (internal) branches versus late (tip) branches of our phylogenetic tree (Figure 5; main text). We assessed the distribution of these isolating alleles over our tree by determining whether the number of isolation loci is linearly proportionate with branch lengths, by assessing specifically whether the number of inferred ‘later’ arising mutations was the same or greater than would be expected given the lengths of the branches on which these mutations occurred. As noted in the main text, however, these tests are substantially underpowered. For example, a minimum ratio of 12:0 of late:early mutations would be required to detect a significant deviation from the linear expectation in our Χ^2^ tests (Table S6), even when the relevant branch lengths are assumed to be estimated without error. When variance in branch lengths (therefore possible error in the estimation of p_early_:p_late_) is considered, the strength of our inferences change according to whether p_early_ is larger than estimated (i.e. the relative length of the internal branch is longer than estimated) or smaller (i.e. the relative length of the internal branch is shorter than estimated) (Table S6). To demonstrate this general expectation, we recalculated our Χ^2^ tests for two extreme cases of error in our estimated branch lengths:

A) the true internal (early) branch length was significantly underestimated in our phylogenetic inference (i.e., true length = est. length+2SD; or p_early_:p_late_ of 0.291:0.709)

B) the true internal (early) branch length was significantly overestimated in our phylogenetic inference (i.e., true length = est. length-2SD; or p_early_:p_late_ of 0.206:0.794)

In the first case, inferences of non-linear accumulation become stronger, whereas in the second case they become weaker (Table S6). This is because increasing the relative length of the internal branch (increasing p_early_) decreases the chance that our observed distribution of sterility-causing mutations (i.e., mostly or entirely on tip branches) would occur via random processes alone. Note that additional cases that consider error on both tip and internal branches, give qualitatively similar results that depend only on whether the net effect is to increase or decrease p_early_. We note, however, that these effects of branch length uncertainty are expected to be quite modest (Table S6), because our estimates of branch length variance are small (Figure S2).

In addition, we can show that our assumption that each QTL is underpinned by a single mutational change, affects our ability to assess whether the number of isolation loci is linearly proportionate with branch lengths. When we relax this assumption, by assuming that 1/3 of ‘late’ QTL are composed of at least 2 loci (as inferred for our co-localized QTL), our results for pollen sterility become much stronger. In particular, both Χ^2^ and binomial analyses indicate that number of ‘late’ pollen mutations is greater than would be expected if isolation mutations accumulate in linear proportion to observed branch lengths (Table S6, S7). Our results for seed sterility do not substantively change (linear model is not rejected). In addition, for completeness, we also performed all of these analyses under an alternative expectation that the number of sterility-causing mutations accumulates in exponential proportion to the observed branch lengths (‘exponential model’). The exponential model is not rejected for either test, for either pollen or seed fertility data (Table S6, S7). Our results for seed sterility do not substantively change (neither linear nor exponential model is rejected).

Overall then, when our assumptions about the number of loci underlying our QTL are relaxed, both Χ^2^ and binomial analyses for testing this relationship reject the ‘linear’ model of accumulation for late-evolving mutations, whereas the exponential model is not rejected for either test (Table S6, S7), although these results depend upon our not having substantially underestimated the length of the internal (early) branch of the three species tree (Table S6). Given the relatively small number of QTL detected, on the basis of these results there is some evidence that later-evolving mutations are significantly more likely to be involved in interactions causing pollen sterility than are earlier-evolving mutations.

***5) Additional sterility phenotypes not associated with tests of allelism***

Our analyses reveal several additional patterns of fertility from both our crossing efforts to create these lines and from analyses of alternative fertility phenotypes/traits in our hybrid lines (Table S2), including the following.

*• Parent-of-origin effects on the success of crosses between introgression lines*

In two of three of our tests of allelism, we found asymmetric effects on crossability. In particular, for *sss1.2* and *sss2.1*, *IL_PP_* did not produce seed when acting as the maternal (seed) parent (i.e. we could not generate *IL_PH_* genotypes). For both *sss1.2* and *sss2.1*, the relevant QTL is a seed fertility locus for which the SP allele has a much larger effect (Table S1). Therefore, it is possible that these *IL_PP_* lines are simply poor maternal parents due to low ovule fertility and/or high rates of early post-fertilization abortion. However, several lines of evidence suggest that asymmetric crossability was more likely due to dysfunctional pollen fertility in the male (sire) genotype. First, the *IL_PP_* genotypes acting as maternal parents are able to successfully produce some seeds from self pollinations (see Figure 3 and 4, main text) indicating at least partial female fertility in these genotypes, that is certainly above that observed when these lines were used as pollen recipients from the *IL_HH_* line. Second, very few (<10%) of attempted hand-pollinations showed evidence of successful fertilization, i.e., swelling of the ovary 3-7 days after pollination. Instead, most crossed flowers eventually senesced and dropped from plants (data not shown). Given this, it is likely that poor (post-mating) pollen performance of *IL_HH_* lines at *sss1.2* and *sss2.1* is responsible for the failure to generate effective seed in *IL_PP_* x *IL_HH_* cross combinations for these 2 QTL, although further crossing studies examining post-pollination pre-fertilization behavior would need to confirm this inference.

*• A maternally-determined locus for seed fertility heterosis*

We observed heterosis for seed fertility specifically in the *IL_HH_* containing *pf9.1*; these lines on average produce nearly 100% more seed than SL parents (Table S2). This increased seed production is also observed in the *IL_HP_* genotype at this locus when *IL_HH_* acts as the seed parent, but not in the reciprocally generated heterospecific *IL_PH_*. This indicates that increased seed production is likely due to a maternal effect specific to the *IL_HH_* seed parent at this locus. The simplest explanation is that this line has higher ovule fertility than the other genotypes to which it was compared, possibly due to quantitative changes in numbers of ovules per ovary in this genotype.

*• Complex effects on pollen fertility at sss2.1*

At *sss2.1*, a pollen sterility locus (*pf2.1*) was previously detected in the SH but not the SP introgression lines (Moyle and Graham 2005, Moyle and Nakazato 2008). Consistent with these previous findings, our analysis indicates that IL_HH_ at this locus has significantly reduced pollen fertility (i.e. contains a pollen sterility locus) but IL_PP_ is not significantly different from the fertile SL parent (Table S2). Our results therefore suggest at least one locus contributes to pollen sterility solely in IL_HH_; when homozygous on the SL genetic background, we estimate the SH allele(s) causes a ~13% reduction in pollen fertility.

Further interpreting results at this locus is made more complex by the finding that the IL_PP_ genotype is statistically intermediate between SL and both other IL genotype classes (Table S2). Combining the SH and SP alleles at this locus therefore fails to significantly ‘rescue’ pollen fertility in the IL_HP_ genotypic class (Table S2). The failure to significantly rescue fertility points to additional complex effects not accounted for by straightforward complementation of a recessive sterility locus.

Several factors could potentially contribute to our observations at this locus. First, we might have insufficient power in the current (and previous) experiment to statistically discern the pollen sterility effect in IL_PP_ individuals at this chromosomal location. Results from our re-analysis of the previous mapping experiment data (Table S8, and see above) is not consistent with a simple failure to pass a stringent statistical cut-off at this chromosomal location in the previous mapping experiment, as we do not detect a PF locus here even under a highly permissive threshold (Table S8). Nonetheless, sterility loci can be environmentally sensitive, and therefore variably observed between different experiments; the IL_PP_ genotype at this locus does have relatively high variation in PF in the current experiment (compare SE for this genotype; Table S2). Therefore, an environmentally sensitive pollen sterility locus at *sss2.1* in IL_PP_ might explain our observations for this specific genotype. Second, the length of the QTL-containing introgressed region in IL_HH_ is larger than in IL_PP_ (see Figure 4A) so that IL_HH_ might carry at least one additional sterility locus not present in the IL_PP_ genotype. If this is the case, the failure to recover fertility in the IL_HP_ genotype here indicates that the SH allele(s) at this sterility locus is at least partially dominant. Finally, additional complex genetic interactions might contribute to these observed fertility patterns. For example, the sterility observed specifically in the heterointrogression genotype might be due to novel deleterious interactions among the three genomes (SL, SH, and SP) that contribute to this genotype. The expected fertility of these ‘tri-genomic’ hybrids will also depend upon the details and importance of potential interactions between loci derived from the two introgression donor species (SH and SP) and the genetic background of the recipient species (SL) in this instance (for example, see discussion of SSNC in Text S1).

In any case, our results are consistent with at least one lineage-specific locus causing reduced pollen fertility in individuals with SH alleles, as well as other possible effects including potentially increased environmental-sensitivity of the SP sterility allele, additional background effects acting in this and the NIL_HP tri-genomic hybrid, and/or additional loci found only in the larger SH introgressed region.

• *Pollen fertility patterns at non-allelic pf9.1 QTL*

For completeness, our complementation analysis also included an additional pair of pollen sterility QTL located on the long arm of chromosome 9 (*pf 9.1*; Table S1), which appeared to be closely adjacent in a comparison between the two mapping experiments (Moyle and Nakazato 2008). As with the other analyses, we generated hybrid introgression lines (i.e., IL_HP_ and IL_PH_ lines) by crossing the IL_PP_ and IL_HH_ lines that each contained the corresponding QTL (hereafter, *pf9.1.1* and *pf9.1.2*) in homozygous state (as in Figure S3). We assayed fertility in these lines, as for the other tests of allelism reported (main text). Because they are not physically colocalized, this pair of pollen sterility QTL is not expected to be allelic.

Consistent with previous QTL analyses, we confirmed that both IL_HH_ and IL_PP_ have reduced pollen fertility (i.e. contain a pollen sterility QTL at *pf9.1.1* and *pf9.1.2*, respectively) in comparison to the fertile SL parent (Table S2). In addition, we observed that reciprocal IL hybrids IL_HP_ and IL_PH_ are intermediate between (and statistically indistiguishable from) both IL_HH_ and IL_PP_ parental introgression lines. Thus, as expected, there is no evidence for allelism in this case.

For this locus, the simplest expectation of fertility relationships might be that alleles from SH and SP should reciprocally complement fertility, as each allele is lineage-specific. As for pollen sterility effects at *sss2.1*, the failure to significantly rescue fertility in heterointrogression genotypes potentially points to additional complex interactions between alleles at different loci in SL, SH, and SP. Indeed, we observed large variation in the fertility of IL_HP_ and IL_PH_ lines (compare SE with other genotypes in this comparison; Table S2) suggesting increased sensitivity to environmental effects in these complex hybrids. A tentative inference is that patterns of sterility are consistent with complex effects of combining loci from three species, an inference that will require identification of the underlying loci for definitive assessment.

**Text S2: Citations**

Moyle LC, Graham EB (2005) Genetics of hybrid incompatibility between *Lycopersicon esculentum* and *L. hirsutum*. Genetics 169: 355-373.

Moyle LC, Nakazato T (2008) Comparative genetics of hybrid incompatibility: Sterility in two Solanum species crosses. Genetics 179: 1437-1453.
